# YOLO-SDA: an innovative YOLOv12-derived model with superior performance in recognizing peanut foliar diseases

**DOI:** 10.3389/fpls.2026.1780712

**Published:** 2026-03-31

**Authors:** Dexu Yang, Jinmiao Chen, Chunyu Wang, Jing Wang, Zhixia Liu, Xilin Zhong

**Affiliations:** 1 College of Engineering, Shenyang Agricultural University, Shenyang, China; 2 College of Engineering, Anhui Agricultural University, Hefei, Anhui, China

**Keywords:** deep learning, lightweight, object detection, peanut leaf diseases, YOLO

## Abstract

**Introduction:**

Manual detection of peanut leaf diseases is plagued by a significant time lag, which frequently enables diseases to develop from isolated, sporadic outbreaks into large-scale epidemics. This delay ultimately leads to substantial regional yield losses in peanut production. Consequently, the precise detection capability of intelligent monitoring equipment is essential for mitigating the risk of large-scale peanut disease outbreaks. Detection algorithms serve as the core technology underpinning intelligent detection devices, highlighting the need for optimized, high-performance algorithms to address this challenge.

**Methods:**

This study takes the YOLOv12 algorithm as the baseline model and proposes an improved model named YOLO-SDA. To enhance the model’s performance while reducing its computational burden, three key modules—StarNet, DySample, and A2C2f_SCSA—are integrated into the original YOLOv12 framework. The integration of these modules is designed to optimize feature extraction, sampling efficiency, and feature fusion, thereby improving the model’s detection accuracy and reducing its resource consumption.

**Results:**

Experimental results demonstrate that the proposed YOLO-SDA model outperforms the baseline YOLOv12 model in both performance and efficiency. Specifically, compared with YOLOv12, the YOLO-SDA model achieves a 44% reduction in parameters, a 38.5% decrease in GFLOPs (giga floating-point operations per second), and a 43.6% reduction in model size. Simultaneously, the model’s detection precision and mAP@0.5–0.95 (mean average precision at intersection over union thresholds from 0.5 to 0.95) are improved by 2.0% and 2.5%, respectively.

**Discussion:**

The superior performance of the YOLO-SDA model confirms the effectiveness of integrating StarNet, DySample, and A2C2f_SCSA modules into the YOLOv12 framework. The significant reduction in parameters, GFLOPs, and model size addresses the practical challenge of deploying intelligent detection algorithms on resource-constrained equipment, making it more suitable for on-site peanut leaf disease monitoring. The concurrent improvement in detection precision and mAP@0.5–0.95 ensures that the model can accurately identify peanut leaf diseases even in complex field environments, providing a reliable technical support for preventing large-scale disease outbreaks and safeguarding peanut yield.

## 1 Introduction

Peanuts are a major oilseed and cash crop worldwide (Feng et al., 2024; Xu et al., 2021, Xu et al., 2023, Xu et al., 2024). China ranks among the leading peanut-producing countries, with a cultivation area of approximately 199 million mu (about 13.3 million hectares), placing it second globally, and a production volume that ranks first worldwide. The condition of peanuts during their growth period is crucial for ensuring yield and quality. Environmental factors such as high temperatures and humidity readily induce leaf diseases in peanuts, leading to reduced yields and diminished quality (Anco et al., 2020; Zhao et al., 2025). The identification and differentiation of peanut leaf diseases are crucial for timely prevention and control. Currently, disease detection primarily relies on manual visual inspection, which has limitations in terms of accuracy and timeliness, making it difficult to precisely control the timing and dosage of pesticide application. Untimely detection of diseases can easily lead to widespread outbreaks, while excessive pesticide use may cause pathogens to develop resistance and result in environmental pollution (Waliyar et al., 2000). Therefore, there is an urgent need to establish efficient detection methods to enable early detection and intervention of diseases, thereby gaining time for timely prevention and control, reducing the severity of disease outbreaks, and effectively safeguarding peanut yields.

Over the past decade, traditional machine learning models for detecting plant diseases have emerged, such as K-Nearest Neighbors (KNN) (Hayit et al., 2024), Support Vector Machine (SVM), Decision Trees (DTs) (Abuhayi and Hajdu, 2025), and Random Forests (RFs) (Alzakari et al., 2024) and Multilayer Perceptron (MLP) (Anandamurugan et al., 2022), These models have played a significant role in plant disease detection, but they also have limitations such as weak generalization capabilities and limited capacity for processing large-scale data. Therefore, there is an urgent need for accurate disease detection models to improve detection precision.

Today, computer vision technologies based on machine learning and deep learning have become a research hotspot and have achieved significant progress. Currently, various methods have emerged for detecting diseases, such as those, Bao et al. (2023) proposed an unmanned aerial vehicle (UAV) remote sensing method based on DDMA-YOLO for effectively detecting and monitoring TLB while reducing the workload and time consumption of this process. Compared with the baseline network, the AP@0.5 of the proposed method increased by 3.8%, and the recall increased by 6.5%. Zhang et al. (2023) performed fine-grained feature learning of diseases was performed using a deep-learning method to achieve tomato disease degree detection. The proposed model achieved an accuracy rate of 95.03% in detecting the severity of tomato diseases and an accuracy rate of 98.25% in identifying the types of tomato diseases. To address the challenges of low efficiency for detecting the cotton verticillium wilt, a lightweight two-stage segmentation model based on improved DeepLabV3+ was developed (Xu et al., 2025a), which can accurately extract diseased leaves and spots in field environments. The number of parameters and floating-point operations in the improved model was minimal, achieving 5.02 M and 27.37 G, respectively. Thai et al. (2025) introduced a drone-based remote sensing and deep neural network model for detecting banana leaf diseases, enabling automated leaf disease detection in large-scale farmlands. The proposed model requires at least 2.09 times fewer parameters and 3.18 times fewer GFLOPs. Yu et al. (2025) proposed a model integrating disease-specific spectral characteristics with physiological parameters, enabling high-precision detection of early-stage ShB through data transformation. The proposed MMCG-MHA model demonstrates significantly superior performance compared to unimodal approaches, achieving a classification accuracy of 94.1667%. This represents improvements of 18.96% and 11.65% over GRU and CNN, respectively. Liu et al. (2024) proposed the improved SEA-YOLOv5 model for peanut kernel quality detection. The enhanced model achieved an accuracy of 98.8%, with parameters totaling only 0.47 million and a single-image detection time of 11.2 milliseconds. Not only does it outperform other models in precision, but it is also suitable for resource-constrained embedded devices such as mobile terminals. Liu et al. (2025) proposed an improved model, SSE-YOLOv5s, for the accurate detection of peanut pod appearance quality. Experimental results show that its parameters are only 6.7% of the original model’s, achieving a frame rate of 115 FPS, with detection accuracy improved by 1.6 percentage points and mAP enhanced by 0.7 percentage points.

The algorithm above has demonstrated effective capabilities in detecting diseases and assessing appearance quality within the agricultural sector, and has also made significant contributions to the detection of leaf diseases in peanuts. For example, Lin et al. (2024) proposed an automated leaf disease detection system. Compared to the original YOLOv8n, the model parameters and FLOPS were reduced by 31.01% and 45.40%, respectively, while achieving an average accuracy of 91.10% and a precision of 89.80%. Lv et al. (2025) proposed an efficient deployment of peanut leaf disease detection models on edge AI devices. Results demonstrated that integrating the ReLU activation function with convolutional operations reduced inference latency by 55.5%. Integrating the EfficientNMS_TRT module further reduced inference latency by 19.6% while increasing frames per second (FPS) by 20.4%. After conversion to NHWC format, model conversion time decreased by 88.7% and inference latency dropped by 32.3%. Feng et al. (2023) proposed an online identification method for peanut leaf diseases based on data balancing algorithms and deep transfer learning to address data distribution skewness. The results demonstrated that the average macro-level accuracy for peanut leaf disease identification reached 0.978, 0.990, and 0.974, respectively. Guo et al. (2024) proposed a novel method for predicting peanut leaf spot disease. Results indicated that compared to standalone CNN and LSTM models, the root mean square error (RMSE) decreased by 0.253 and 0.204, respectively, while the coefficient of determination (R²) increased by 0.155 and 0.122, respectively.

The above research findings lay an important foundation for the development of intelligent detection devices for crop diseases and other applications. However, certain limitations exist, such as insufficient dataset scale and diversity, a large number of parameters, and a lack of applicability to intelligent detection devices. Therefore, this study employs the latest YOLOv12 as a baseline to explore lightweight methods for peanut leaf disease detection that reduce parameter counts while maintaining detection accuracy, thereby achieving a new detection model. The contributions of this paper are summarized as follows:

1. Targeted lightweight backbone for small lesion detection: We propose a hybrid backbone that integrates StarNet’s star operation into the YOLOv12 architecture. This is the first attempt to leverage element-wise multiplication for local feature enhancement within a ViT-based detection framework, specifically designed to improve the detection of tiny, early-stage peanut leaf spots.
2. Dynamic upsampling for fine-grained detail reconstruction: We introduce DySample, a learnable dynamic upsampler, to replace static interpolation in the Neck network. This innovation adaptively reconstructs the detailed structure and contours of small lesions during feature fusion, effectively mitigating the information loss that leads to high missed detection rates.
3. Synergistic attention for complex background suppression: We design a novel A2C2f_SCSA module that embeds Spatial and Channel Synergistic Attention (SCSA) into the feature fusion network. Unlike conventional attention, this module simultaneously suppresses background noise (e.g., soil, overlapping leaves) and enhances disease-relevant features, significantly reducing false positives in complex field environments.
4. Extreme lightweighting for embedded deployment: Through the synergistic integration of these three modules, the resulting YOLO-SDA model achieves a 44% reduction in parameters and 43.6% reduction in model size compared to YOLOv12, while improving mAP@0.5-0.95 by 2.8%, making it highly suitable for real-time inference on resource-constrained edge devices such as the Raspberry Pi.

## 2 Materials and methods

### 2.1 Materials

**Figure 1:**
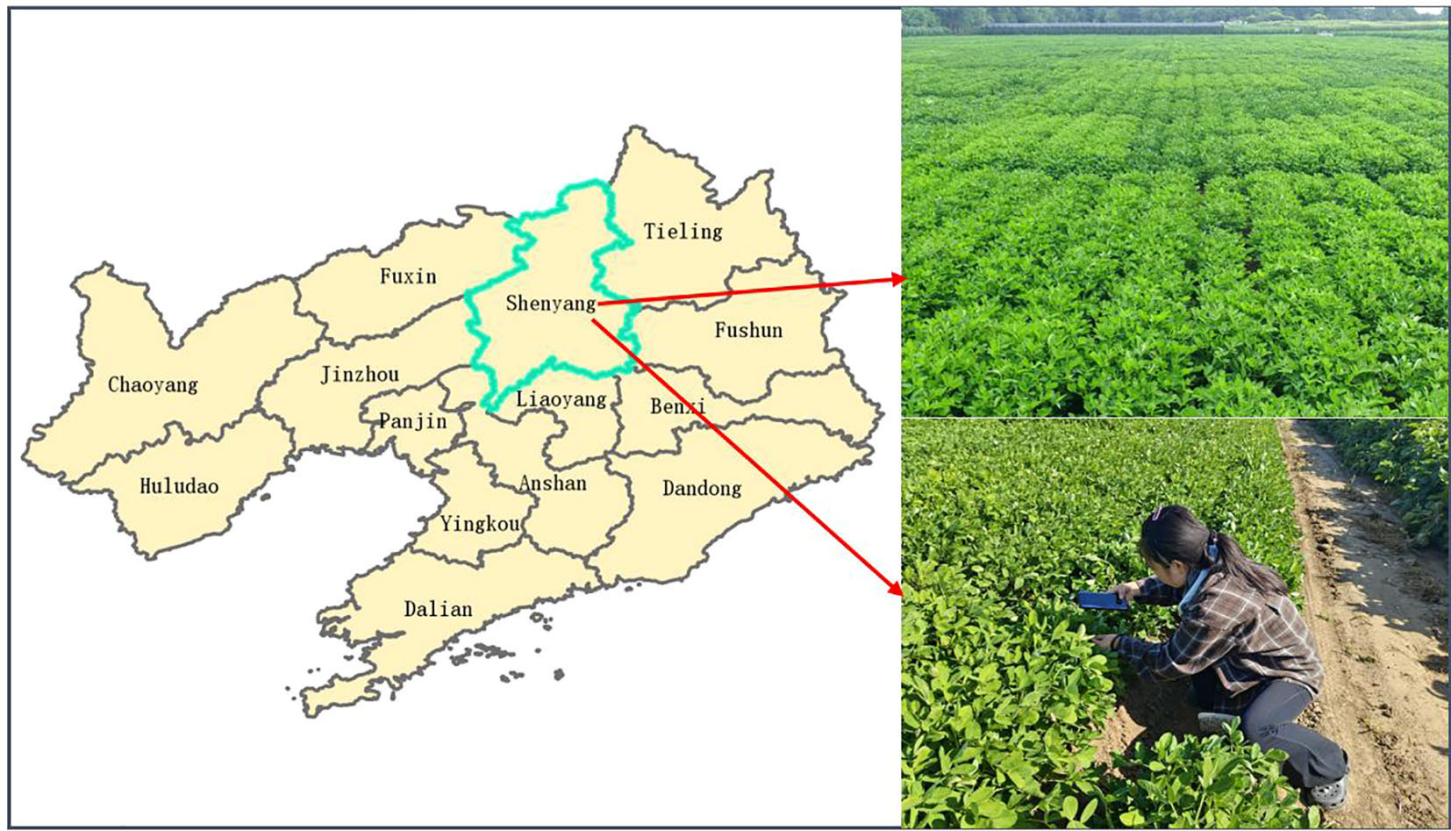
Image acquisition site at the peanut experimental base of the Peanut Research Institute, Shenyang Agricultural University.

**Figure 2:**
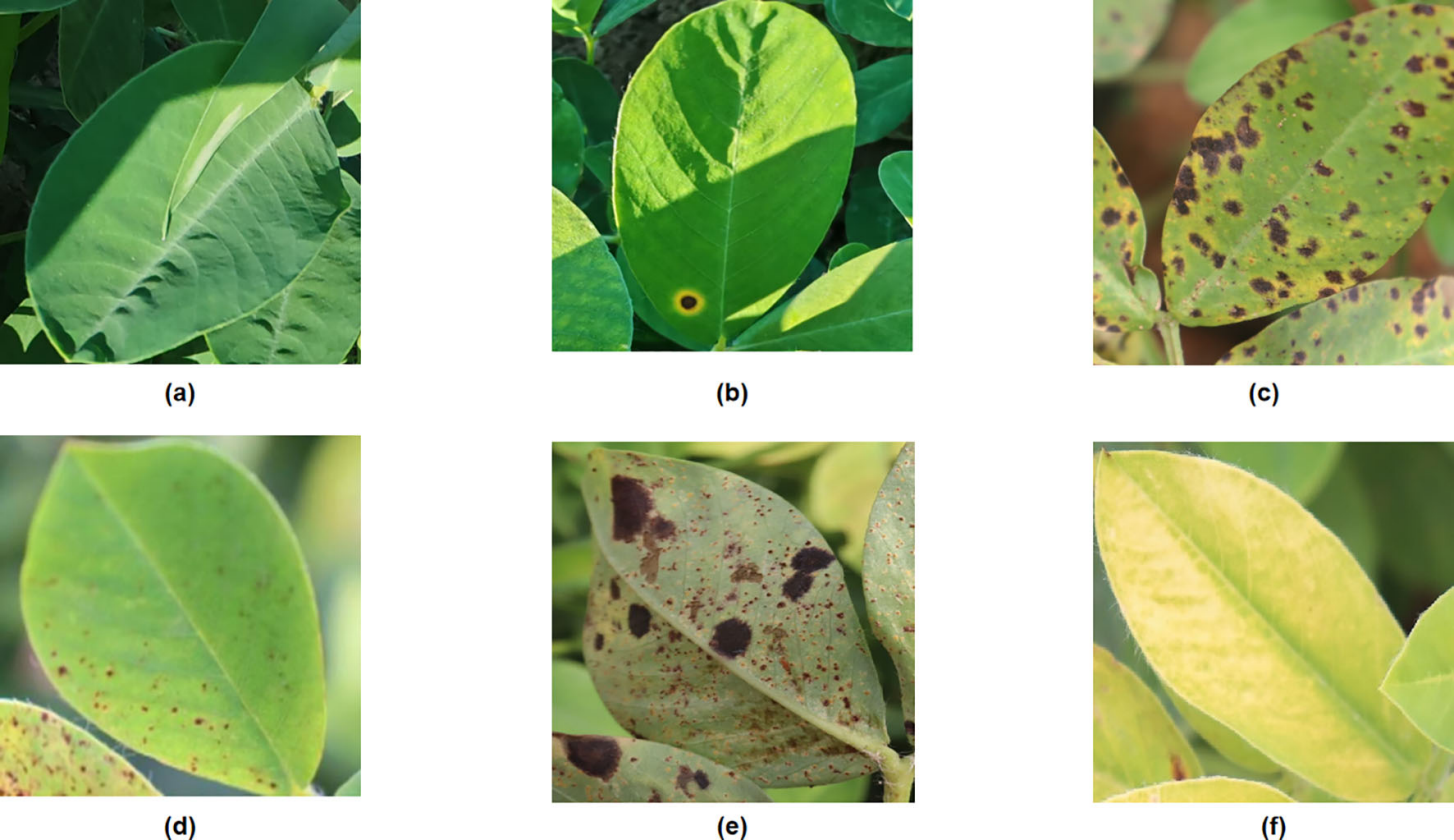
Comparison of samples across different categories: **(A)** Healthy, **(B)** Early leaf spot disease, **(C)** Advanced leaf spot disease, **(D)** Early rust disease, **(E)** Rust disease, **(F)** Nutrient deficiency.

The original images of peanut leaf diseases in this experiment consist of two parts: images collected in the field at the Peanut Research Institute’s experimental base of Shenyang Agricultural University and images collected online. The Peanut Research Institute of Shenyang Agricultural University is located at 120 Dongling Road, Shenyang City, Liaoning Province, China (41.82N, 123.56E). The actual image acquisition device used was the Huawei HONIR 100Pro smartphone with a resolution of 4096×3072 pixels. Images were captured at a distance of 0.2 to 0.5 meters from peanut leaves, yielding a total of 472 images (**Figure 1**). Due to the limited variety of diseases present at this peanut trial site, the number of collected image samples was insufficient. Therefore, online image acquisition was employed to capture diverse disease characteristics under complex field conditions. The web scraping tool is implemented using the Python programming language, with core dependencies on the requests library for sending HTTP requests, the json library for parsing returned data, and file system operations. Batch crawling is achieved by inputting keywords via a script. The script employs urllib.parse.quote to URL-encode keywords and sets a dynamic request interval of 0.9 seconds per request, collecting 1,550 images online. By automatically filtering images collected from the web using scripts, we excluded non-RGB formats, blurry images with resolutions below 200×200 pixels, samples with severe background interference, and duplicates. This process yielded 1,000 valid images from the web collection. A total of 1,472 effective images of peanut leaf diseases were collected through field surveys and online sources. The dataset of 1,472 images was uniformly resized to 640x640 pixels and categorized into six classes (**Figure 2**) (Sun et al., 2025): healthy leaves (256 images), early-stage leaf spot disease (249 images), late-stage leaf spot disease (243 images), early-stage rust disease (239 images), late-stage rust disease (236 images), and nutrient deficiency (249 images).

**Figure 3:**
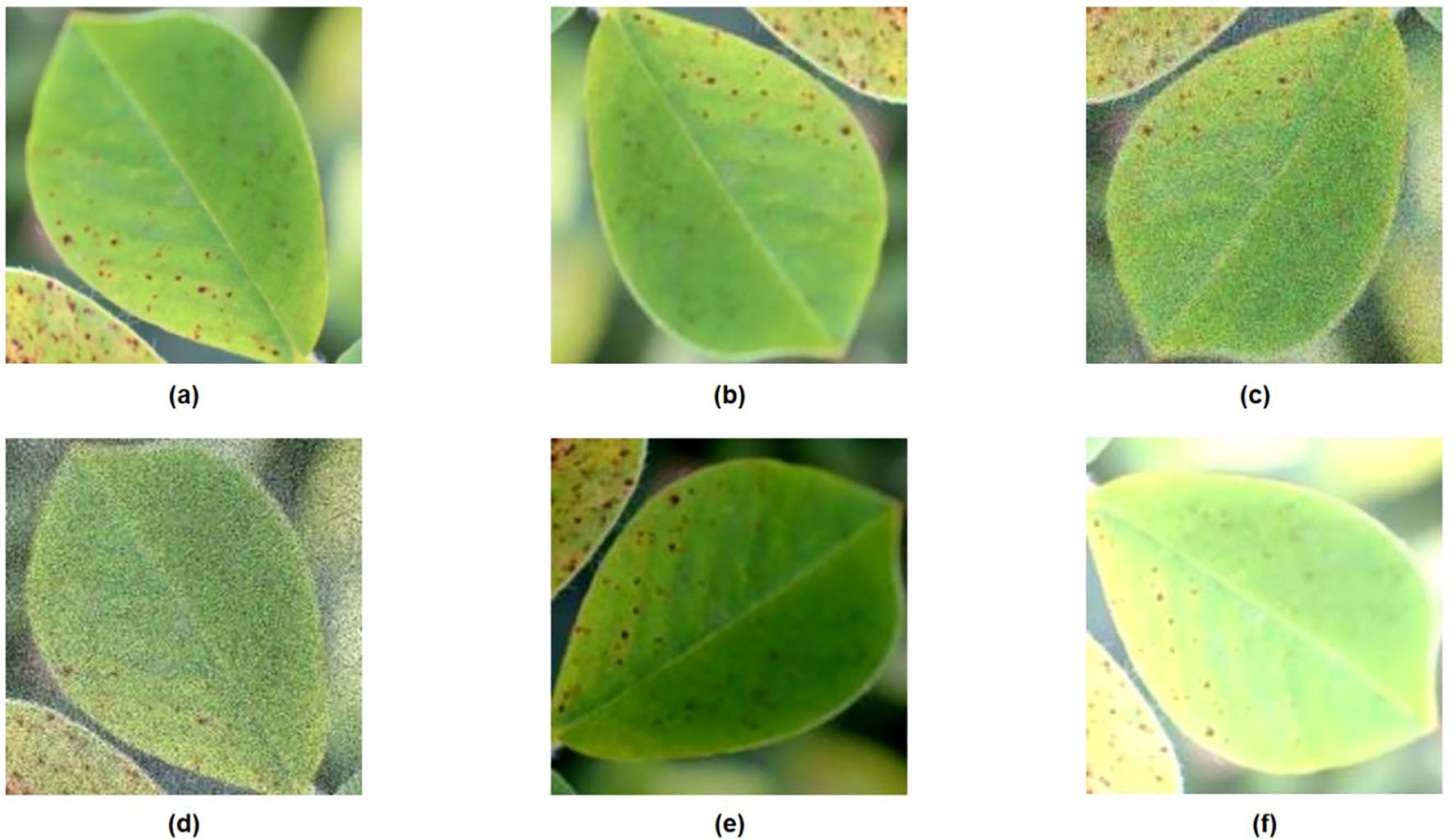
Enhanced images of leaf diseases: **(A)** Original image, **(B)** Inverted image, **(C)** Gaussian noise, **(D)** Salt-and-pepper noise, **(E)** Brightness adjustment, **(F)** Exposure enhancement.

The collected dataset of 1,472 images underwent manual annotation and augmentation for preprocessing. Use the LabelImg tool to annotate disease images with rectangular labels. Classify them by creating different disease labels and generating a TXT-type annotation file. Given that the dataset size remains insufficient for network model training and the image backgrounds lack sufficient diversity, this experiment employed five data augmentation techniques—random rotation, Gaussian noise, salt-and-pepper noise, brightness adjustment, and exposure adjustment—to enhance the dataset. Ultimately, the dataset was expanded from 1,472 images to 7,360 images (**Figure 3**) (**Table 1**). The annotated and augmented data were divided into a training dataset (5,150 images), a validation dataset (1,470 images), and a test dataset (740 images) in a 7:2:1 ratio.

**Table 1:** Details of the peanut leaf disease dataset.

Disease category	Original images	After augmentation
Healthy	256	1280
Early Leaf Spot	249	1245
Late Leaf Spot	243	1215
Early Rust	239	1195
Late Rust	236	1180
Nutrient Deficiency	249	1245
Total	1472	7360

### 2.2 Methods

#### 2.2.1 YOLOv12 network model

Using the YOLOv series models for detecting leaf diseases in peanuts, the detection results are shown in **Table 2**. After a comprehensive evaluation of all performance metrics, YOLOv12 emerges as the relatively optimal choice. In terms of detection accuracy, the YOLOv12 achieves an mAP@0.5-0.95 of 89.7%, with a Precision of 97.6% and a Recall of 98.7%, indicating both precise disease category identification and reduced missed detection rates. In terms of model lightweight and computational complexity, YOLOv12 has only 2.5 MB of parameters and 6.5 GFLOPs, with a model size of 5.5 MB. This indicates that YOLOv12 has low hardware capacity requirements during training, thereby reducing training costs. In terms of deployment, it is easier to run on both local and edge devices, offering greater practicality and cost-effectiveness. Compared to other versions, such as YOLOv8, which boasts the highest recall rate, it falls short of YOLOv12 in core metrics, including accuracy, lightweight design, and computational efficiency. Overall, YOLOv12 better aligns with the practical requirements for detecting leaf diseases in peanuts.

**Table 2:** Comparative results of detection performance for different YOLO versions.

Methods	Parameters /MB	FLOPs /G	Model size /MB	Precision /%	Recall /%	mAP@0.5-0.95 /%
YOLOv5	7.1	16.3	14.4	82.1	75.5	73.1
YOLOv7	9.3	26.7	19.0	76.1	77.0	72.2
YOLOv7-tiny	6.0	13.2	12.3	79.0	77.6	71.8
YOLOv8	3.0	8.1	6.0	88.3	92.0	78.8
YOLOv10	2.7	8.4	5.8	96.7	97.5	84.9
YOLOv11-tiny	2.5	6.4	5.4	97.0	98.1	87.2
**YOLOv12**	**2.5**	**6.5**	**5.5**	**97.6**	**98.7**	**89.7**

*Results in **Table 2** are from a single representative run. For statistical analysis over five runs, please refer to **Table 8**.

The bold values represent the optimal results of each corresponding evaluation metric.

**Figure 4:**
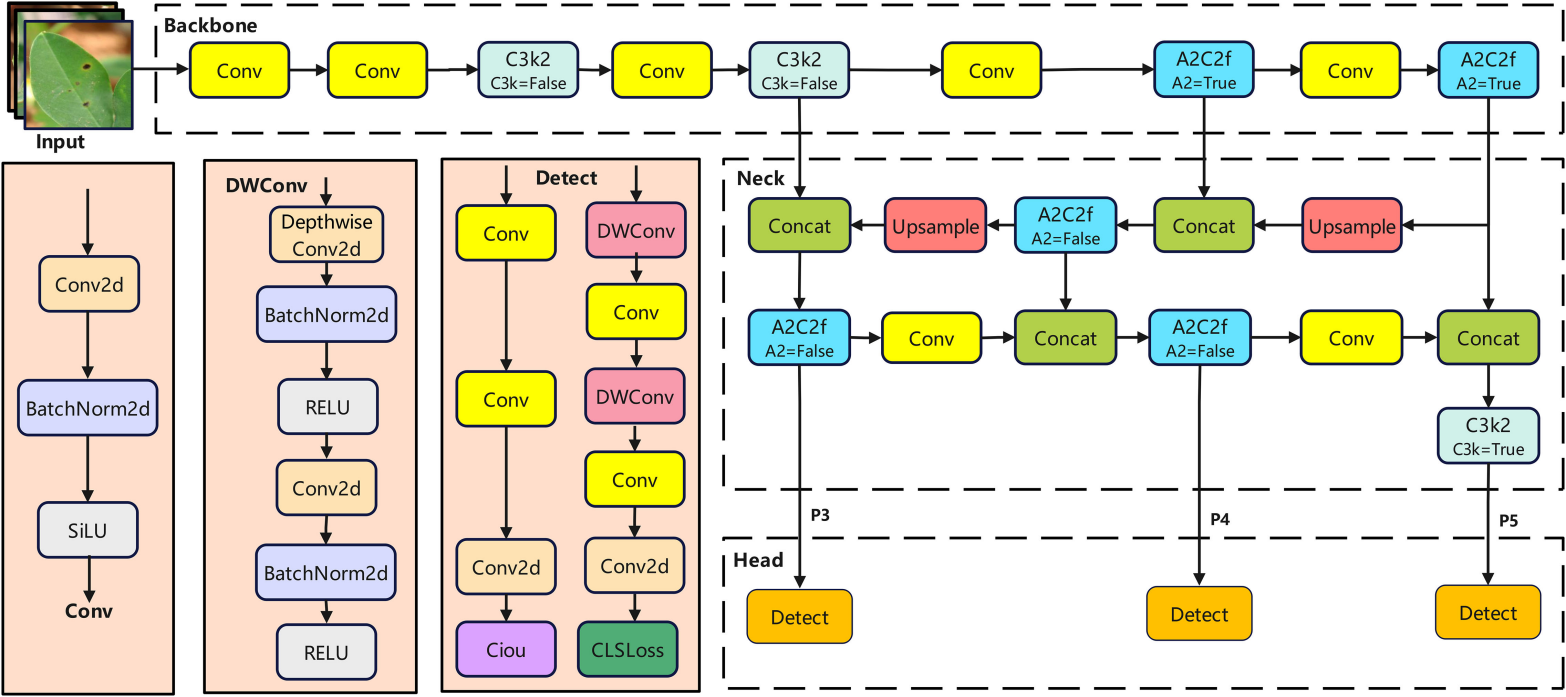
YOLOv12 network structure diagram.

YOLOv12 is a real-time detection technology jointly developed by teams from New York University, the University of Chinese Academy of Sciences in Beijing, and the University at Buffalo. Its architecture consists of a backbone network, a neck network, and a head network (**Figure 4**). This framework maintains high-speed inference while fully leveraging its performance advantages. The R-ELAN network architecture employs multiple Conv blocks, C3k2, and A2C2f modules to perform feature extraction while maintaining lightweight parameters. The Conv modules incorporate Conv2d, batch normalization, and SiLU activation functions. Feature enhancement is achieved through block-level residual connections and adaptive scaling strategies, complemented by 7×7 large-kernel separable convolutions to expand the receptive field. This approach strengthens the model’s ability to extract auxiliary features and localize small objects in complex scenes (Tian et al., 2025). The Neck module incorporates Concat, Upsample, and A2C2f components to fuse and refine features extracted by the Backbone. It combines regional attention with R-ELAN, then integrates feature information across different levels through upsampling and concatenation operations to enhance feature representation. The Head is composed of multiple detect modules and is responsible for the final object detection task, outputting the detected object categories and location information.

#### 2.2.2 StarNet

YOLOv12 employs the Vision Transformer as its backbone network. Despite optimization attempts through designs such as A² and R-ELAN, it still faces challenges, including high computational complexity, inadequate modelling of small objects, and weaker local feature extraction capabilities compared to CNNs. To mitigate the high computational cost of the original YOLOv12’s Vision Transformer (ViT)-based backbone and to enhance the extraction of fine-grained local features critical for detecting small or indistinct lesions, this paper incorporates the lightweight StarNet neural network model into its backbone architecture. This approach enhances local details and positional priors through convolutions, reduces computational costs, and optimizes hardware adaptability. It also better balances the demands for accuracy, real-time performance, and deployment efficiency in peanut leaf spot detection tasks.

**Figure 5:**
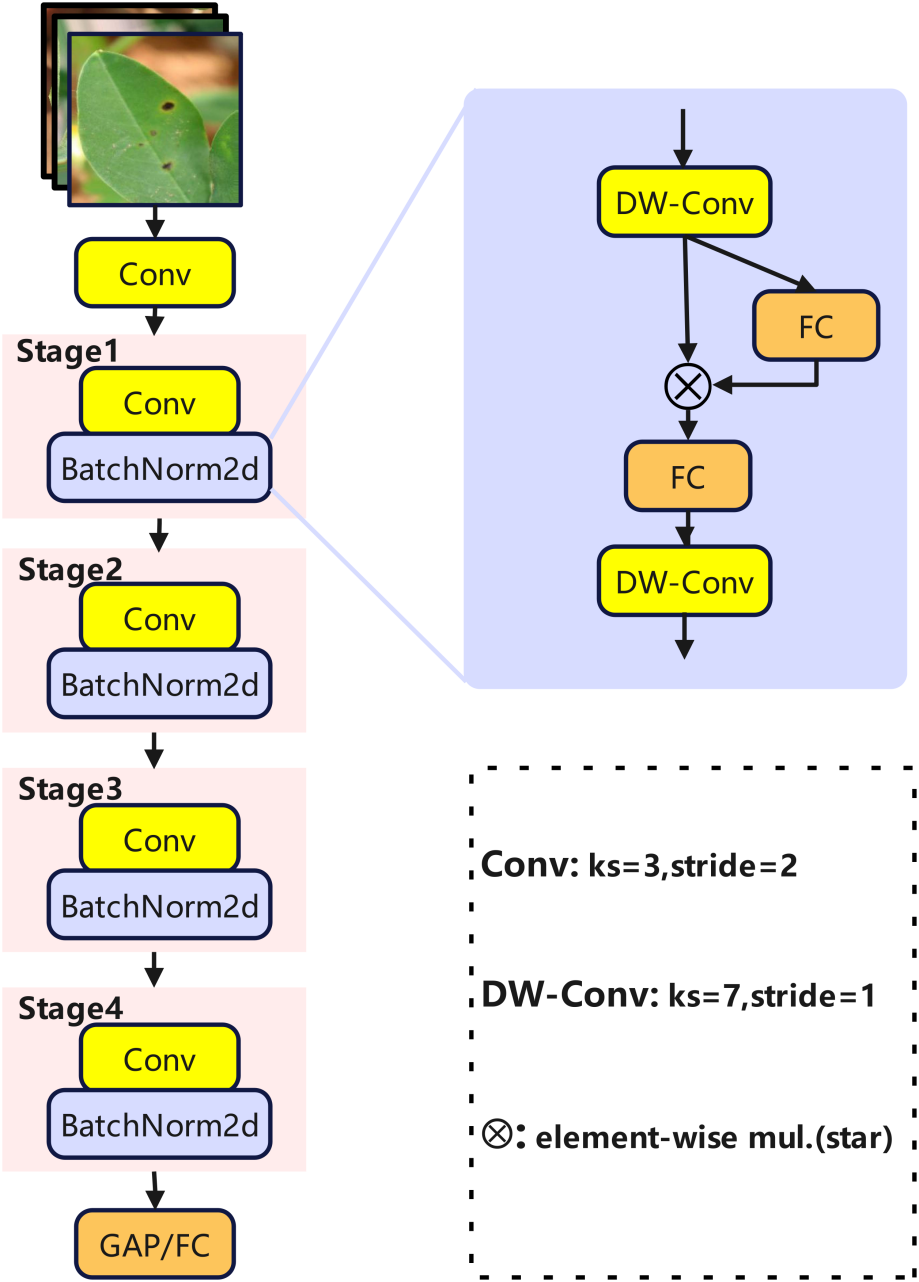
StarNet structure diagram.

StarNet (Ma et al., 2024) was first introduced in 2023 as a lightweight neural network model, primarily optimized for real-time computer vision tasks on edge devices. It aims to balance model accuracy and computational efficiency through an innovative architecture. StarNet is a four-stage hierarchical architecture (**Figure 5**).

Let the input image be represented as $X_{0}\in R^{3\times H_{0}\times W_{0}}$, The initial convolution uses a convolution kernel of $W_{\text{init}}\in R^{C_{\text{init}}\times 3\times 3\times 3}$, and offset ${\text{b}}_{init}\in R^{C_{init}}$, According to Equation 1, the output is:

(1) $$Z_{\text{init}}=W_{\text{init}}X_{0}+b_{\text{init}}$$

After processing the initial feature map through the BN and ReLU activation functions σ according to Equation 2, the result provides a foundation for subsequent processing:

(2) $$X_{\text{init}}=\sigma (\text{BN}(Z_{\text{init}}))$$

To achieve spatial downsampling and channel adjustment, each stage incorporates a convolutional block. For the input features $X_{\text{in}}\in R^{C_{\text{in}}\times H\times W}$ apply a convolution kernel $W_{\text{conv}}\in R^{C_{\text{out}}\times C_{\text{in}}\times 3\times 3}$ and bias ${\text{b}}_{\text{conv}}$ according to Equation 3, performing convolution with a stride of 2:

(3) $$Z_{\text{conv}}=W_{\text{conv}}{*}_{s=2}X_{\text{in}}+b_{\text{conv}}$$

Perform normalization and activation processing according to Equation 4:

(4) $$X_{\text{conv}}=\sigma (\text{BN}(Z_{\text{conv}}))$$

Reduce the spatial dimensions to $\frac{H}{2}\times \frac{W}{2}$. The core of feature enhancement lies in Star Blocks, a module that integrates local spatial features with cross-channel information through a branching design. According to Equation 5, input $X_{\text{s}}\in R^{C_{s}\times H_{s}\times Ws}$, Branch 1 employs a 7×7 depth convolutional layer with a kernel $W_{\text{dw}}\in R^{C_{s}\times 1\times 7\times 7}$ to capture cell-level granularity in space:

(5) $$X_{\text{dw}}=W_{\text{dw}}{*}_{\text{dw}}X_{\text{s}}$$

Branch 2 employs a 1×1 depthwise convolution with a kernel size of $W_{\text{fc}}\in R^{C_{s}\times C_{s}\times 1\times 1}$ and a bias of ${\text{b}}_{\text{fc}}$ to achieve cross-channel interactions according to Equation 6:

(6) $$X_{\text{fc}}=W_{\text{fc}}*X_{\text{s}}+b_{\text{fc}}$$

The two branches are merged through element-wise multiplication using Equation 7:

(7) $$X_{\text{mul}}=W_{\text{dw}}⊗X_{\text{fc}}$$

Simultaneously adding residual connections to maintain the information flow, the final output of Star Blocks is generated according to Equation 8:

(8) $$X_{\text{star}}=X_{\text{mul}}+X_{\text{s}}$$

Apply GAP compression to reduce the spatial dimension of the output from Stage 4 according to Equation 9:

(9) $$X_{\text{gap}}=\text{GAP}(X_{\text{final}})$$

Through end-to-end collaborative optimization, StarNet enhances feature extraction capabilities while maintaining a lightweight design by leveraging multi-scale feature fusion and nonlinear interactions.

#### 2.2.3 DySample

**Figure 6:**
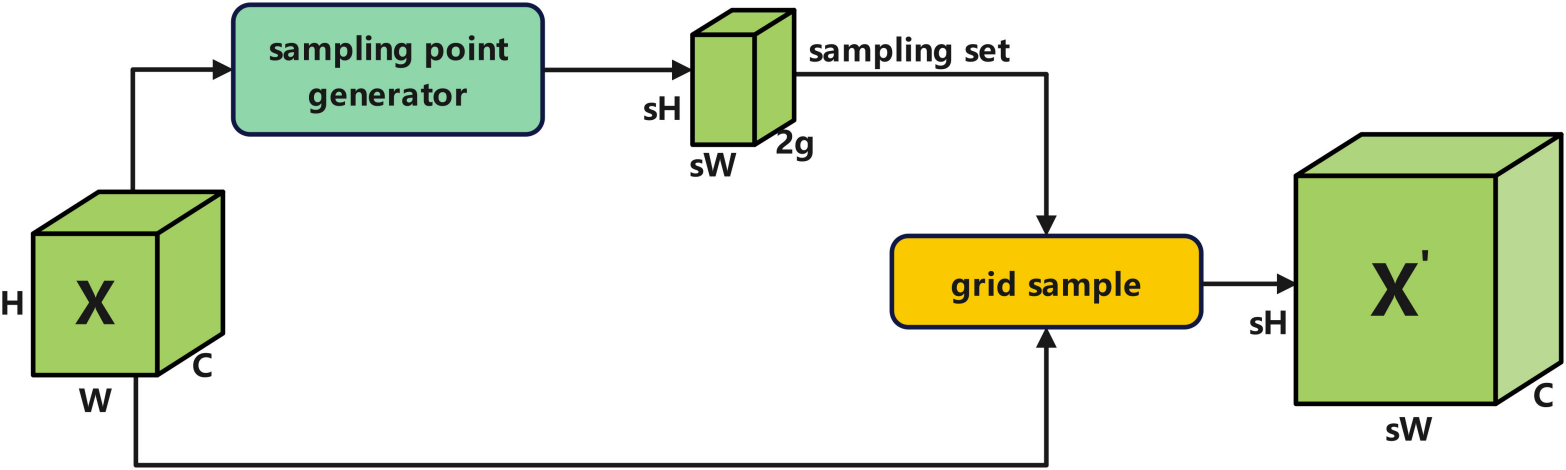
Structural diagram of Dy sample module.

**Figure 7:**
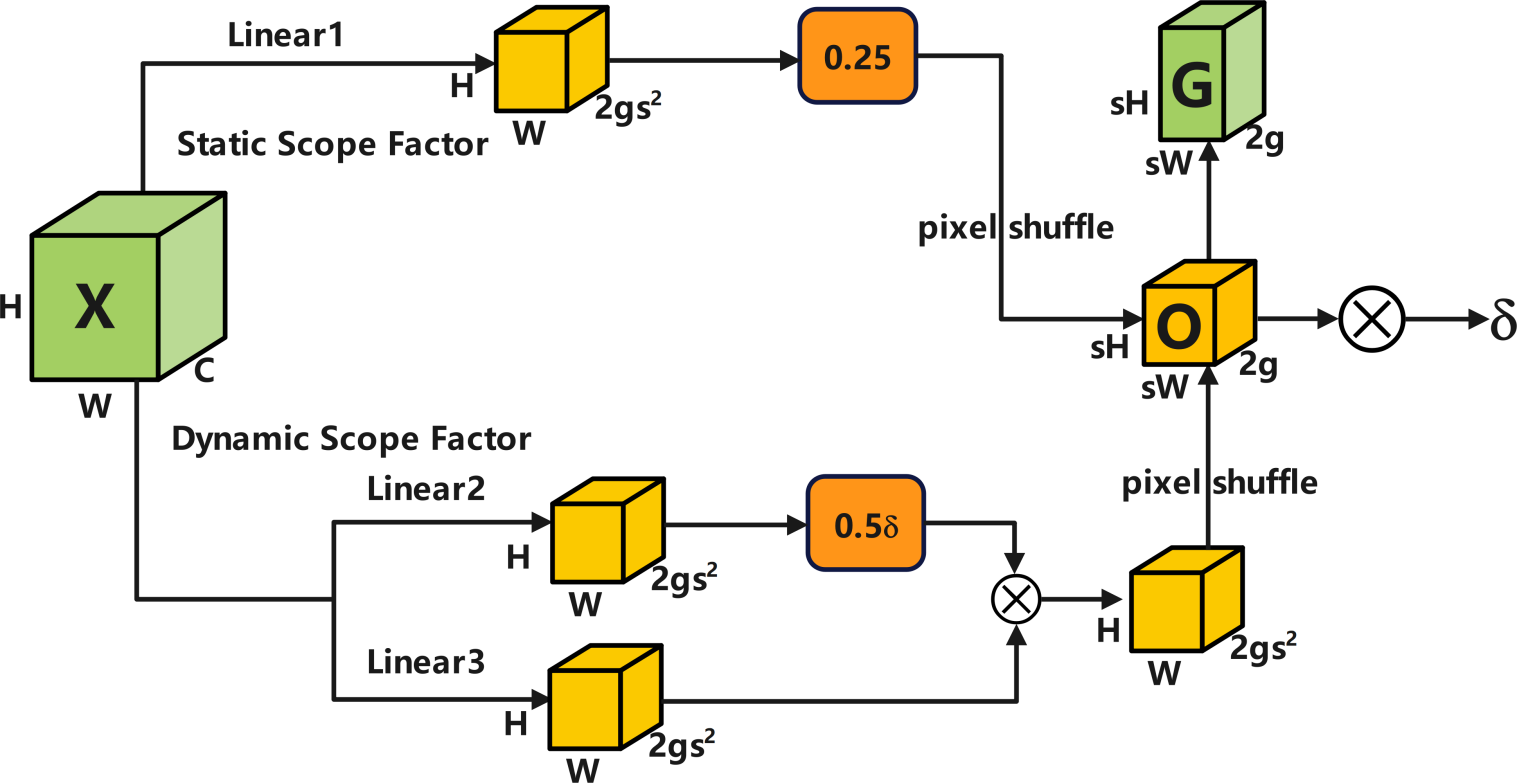
Sampling point generator based on static scope factor and dynamic scope factor.

The nearest-neighbor interpolation used for upsampling in YOLOv12 may lead to issues such as insufficient capture of small target features in leaf diseases. To address this, we incorporate the DySample dynamic upsampler (Liu et al., 2023) into the Neck network. Its dynamic offset mechanism allows for more accurate reconstruction of these details, which are often lost with conventional upsampling. It achieves efficient feature resampling while maintaining low computational cost by synergistically adjusting the sampling offset through static and dynamic range factors (**Figures 6**, **7**) (Pan et al., 2025).

Let the input feature be $X\in R^{H\times W\times C}$, where H and W denote spatial dimensions, C represents the number of channels, and the scaling factor is set to s. The target output size is sH×sW, and the grid dimension is g. The dynamic range factor branch generates the baseline sampling offset to ensure the uniform distribution of sampling points. Input feature X undergoes the following transformation via Equation 10:

(10) $$X_{\text{lin}1}=\text{Linear}(X)\in {\mathbb{R}}^{H\times W\times 2gs^{2}}$$

Equation 11 undergoes spatial rearrangement with a Pixel Shuffle scaling factor of 0.25, yielding the static offset:

(11) $$O_{\text{static}}=\text{ PixelShuffle}(X_{\text{lin}1})\in {\mathbb{R}}^{sH\times sW\times 2g}$$

The dynamic range factor branch adaptively adjusts the offset based on input content to mitigate sampling point overlap. Equation 12 first inputs X into two linear layers, Linear2 and Linear3, respectively, yielding:

(12) $$X_{\text{lin}2},X_{\text{lin}3}\in {\mathbb{R}}^{H\times W\times 2gs^{2}}$$

Equation 13 performs element-wise multiplication on both operands and introduces a dynamic scaling factor σ:

(13) $$X_{\text{scale}}=0.5\;\sigma \cdot (X_{\text{lin}2}⊗X_{\text{lin}3})$$

After undergoing Pixel Shuffle reordering, the dynamic offset is output according to Equation 14:

(14) $$O_{\text{dynamic}}=\text{ PixelShuffle}(X_{\text{scale}})\in {\mathbb{R}}^{sH\times sW\times 2g}$$

Add the static and dynamic offsets together to obtain the final sampling offset via Equation 15:

(15) $$O=O_{\text{static}}+O_{\text{dynamic}}\in {\mathbb{R}}^{sH\times sW\times 2g}$$

Overlay this offset with the preset grid $G\in R^{s^{H}\times s^{W}\times 2\text{g}}$ to construct the sampling point set Sampling Set=G+O. Finally, the input features are sampled using grind_sample, and the upsampled results are obtained according to Equation 16:

(16) $$X'=\text{ grid}(X,\text{SamplingSet})\in {\mathbb{R}}^{sH\times sW\times C}$$

Therefore, replacing the UpSample layer with DySample in this paper significantly enhances the quality of multi-scale feature fusion with minimal computational overhead.

#### 2.2.4 A2C2f_SCSA

**Figure 8:**
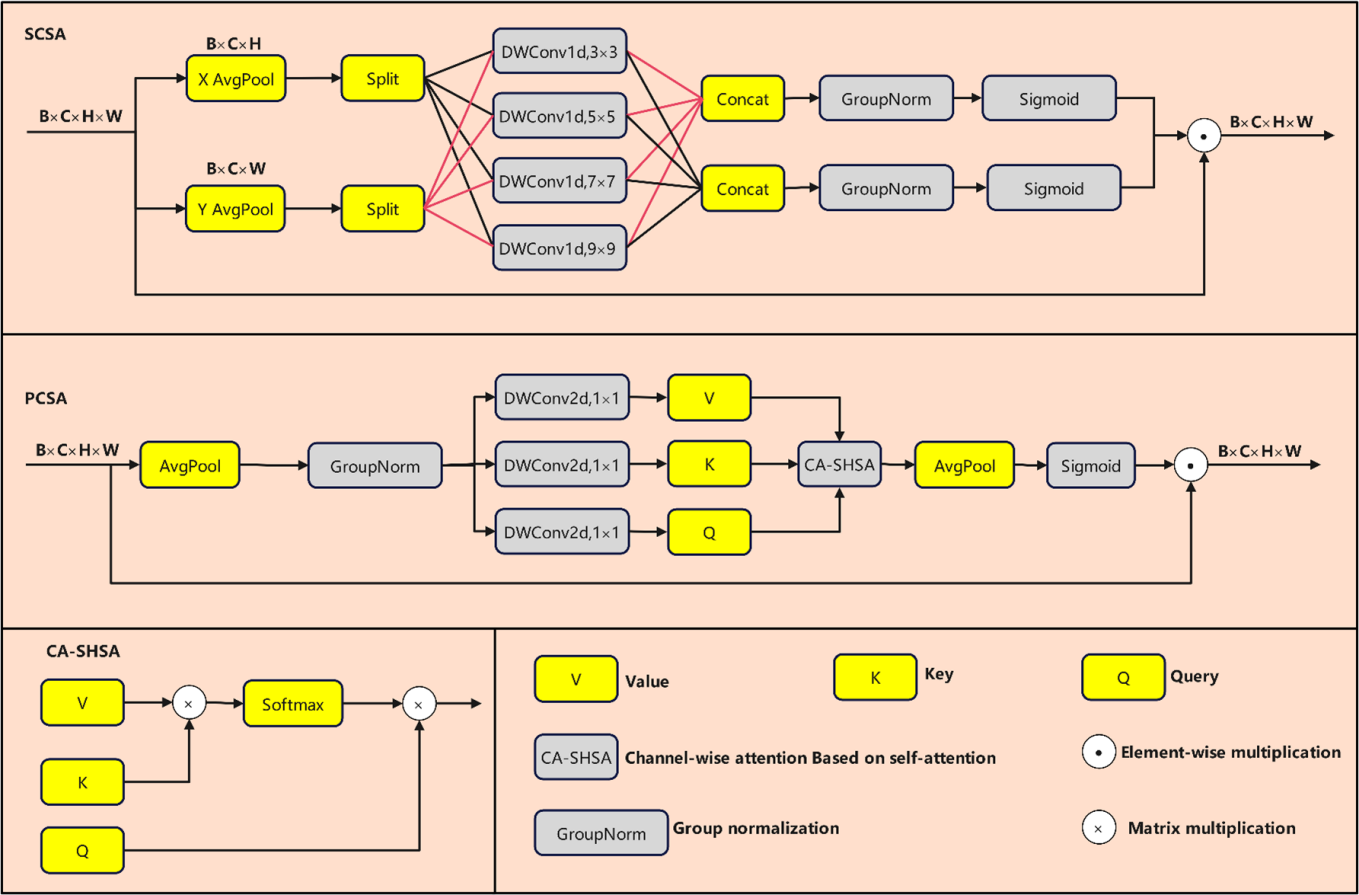
SCSA structure diagram.

The Neck component of YOLOv12 processes features extracted by the Backbone for further refinement before passing them to the output layer for object detection, potentially interfering with the model’s focus on disease features. However, in complex agricultural environments with varying lighting conditions, significant background noise may persist. To address this, the Spatial and Channel Synergistic Attention (SCSA) attention mechanism (Liu et al., 2021) is introduced (**Figure 8**).

Let the input feature map be denoted as $X\in R^{B\times C\times H\times W}$, where B represents the batch size, C denotes the number of channels, and H and W denote the spatial dimensions. The SCSA module consists of two parallel pathways: spatial collaborative attention and channel collaborative attention. Spatial collaborative attention first inputs X through GAP to compress spatial information, yielding $X_{\text{pool}}\in R^{B\times C\times 1\times 1}$. This output is then replicated into three copies via a Split operation and fed into DWConv1d layers with kernel sizes of 3×3, 5×5, and 7×7, respectively. The formula is given in Equation 17:

(17) $$F_{3}=DWC{\text{onvld}}_{3\times 3}(X_{\text{poo}l}),F_{5}=DWC{\text{onvld}}_{5\times 5}(X_{\text{pool}}),F_{7}=DWC{\text{onvld}}_{7\times 7}(X_{\text{pool}})$$

Concatenate the features from the three scales along the channel dimension. Subsequently, apply GroupNorm and the Sigmoid function to generate the spatial attention weights *W_s_*, as defined in Equation 18:

(18) $$W_{S}=\sigma (\text{GroupNorm}(\text{Concat}(F_{3},F_{5},F_{7})))$$

Where σ denotes the sigmoid activation function. ultimately, the spatial pathway outputs are obtained by element-wise multiplication of the original feature map with the spatial weights: $X_{s}=X\;⊗W_{s}$.

Channel-wise attention involves processing input X through GAP and GroupNorm, then generating K and V via two independent 1×1 convolutions, where $K=DWConv2{\text{d}}_{1\times 1}(G\text{roup }N\text{orm}(A\text{vg}P\text{ool}(X)))$ and *V* $=DWConv2{\text{d}}_{1\times 1}(G\text{roup }N\text{orm}(A\text{vg}P\text{ool}(X)))$,Q is obtained from X via GroupNorm to yield Q=Group Norm(X). The channel self-attention calculation is shown in Equation 19:

(19) Achannel= softmax(QK⊤), XC'=AchannelV

To preserve the spatial structure while introducing a one-dimensional depth-wise convolutional branch with a 9×9 kernel, its output is fused with the channel attention weights to generate the channel attention weights $W_{C}$, as shown in Equation 20:

(20) $$W_{C}=\sigma (\text{GroupNorm}(\text{DWConv}1{\text{d}}_{9\times 9}(X_{\text{pool}})))$$

Channel output is $X_{C}=X_{C}^{'}\;⊗W_{C}$.

The final output of the SCSA module is $X_{\text{out}}=X_{s}+X_{c}$, the sum of the results from two pathways, enabling effective coordination between spatial and channel information.

**Figure 9:**
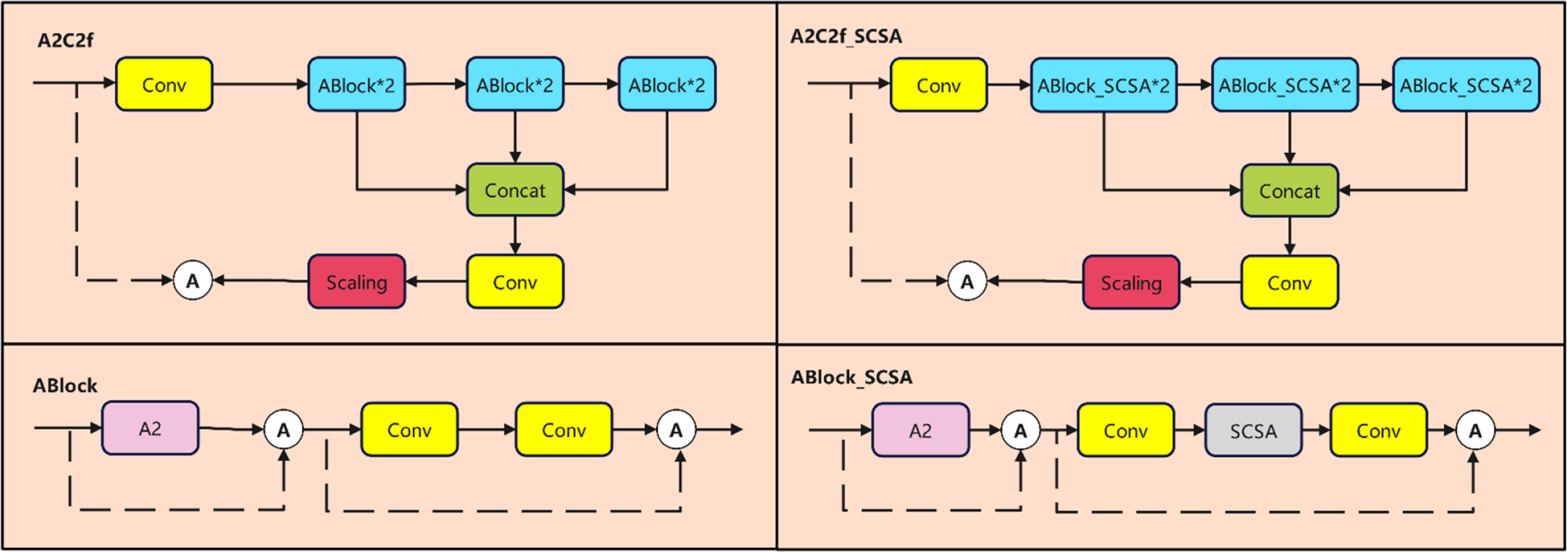
Structure diagram of A2C2F and A2C2f_SCSA.

To effectively embed SCSA into the Neck layer, this paper designs the C2f with Spatial and Channel Synergistic Attention (A2C2f_SCSA) module to replace the original A2C2f module (**Figure 9**). The core improvement lies in replacing the base ABlock module with ABlock_SCSA, which integrates SCSA.

Let the module input be *X*_in_. The forward propagation process of A2C2f_SCSA is expressed by Equation 21:

(21) $$\begin{array}{l} X_{1}=Conv(X_{in})\\ X_{2}=ABlock\_SCSA(ABlock\_SCSA(X_{1}))\\ X_{3}=ABlock\_SCSA(ABlock\_SCSA(X_{1}))\\ X_{\text{ca}t}=Conv(X_{1},X_{2},X_{3})\\ X_{\text{ou}t}=Conv(Scaling(X_{\text{ca}t})) \end{array}$$

Specifically, ABlock_SCSA builds upon the original ABlock architecture by embedding an SCSA attention module between the A2 submodule and the convolutional layer, with the information flow being Y=SCSA(A2(*C*onv (X))).This design ensures that features undergo continuous synergistic enhancement across both spatial and channel dimensions during transformation. Consequently, it maximizes suppression of background noise while preserving and highlighting key features associated with diseased areas—all without significantly increasing computational load.

#### 2.2.5 YOLO-SDA network architecture

To optimize the detection performance of YOLOv12 for peanut leaf diseases in complex agricultural settings, this paper systematically enhances the original model across three dimensions: feature extraction, feature fusion, and feature enhancement. The goal is to improve the model’s feature discrimination capability while preserving its lightweight characteristics.

First, the standard convolutional layers in the original YOLOv12 backbone exhibit significant computational redundancy, and their static weights struggle to adaptively focus on lesion features (Wei et al., 2025). To address this, we introduce the lightweight StarNet module to reconstruct the backbone network. StarNet achieves stronger nonlinear feature representation through element-wise multiplication and residual connections within a dual-branch architecture (local spatial feature extraction and cross-channel interaction). Its core operations can be expressed as $X_{star}=(W_{\text{dw}*\text{dw}}X_{s})⊗(W_{fc*}X_{s})+X_{s}$, This design reduces the number of parameters while enhancing the model’s sensitivity to lesion texture through feature interaction, fundamentally lowering computational complexity and improving feature specificity; Secondly, the UpSample process in the Neck layer sampling procedure is replaced by DySample. This mechanism enables sampling points to dynamically adjust based on input feature content, thereby more accurately reconstructing the detailed structure and contours of lesions during upsampling. This effectively mitigates the loss of fine details in small target lesions; Finally, to suppress interference from complex backgrounds and enhance discrimination between similar targets, we designed the A2C2f_SCSA module at the end of the backbone network and within the feature fusion network. The core of this module involves embedding SCSA into the feature stream. Meanwhile, DySample enhances the capture of small targets and fine-grained details, thereby reducing both false negatives and false positives in complex scenes.

**Figure 10:**
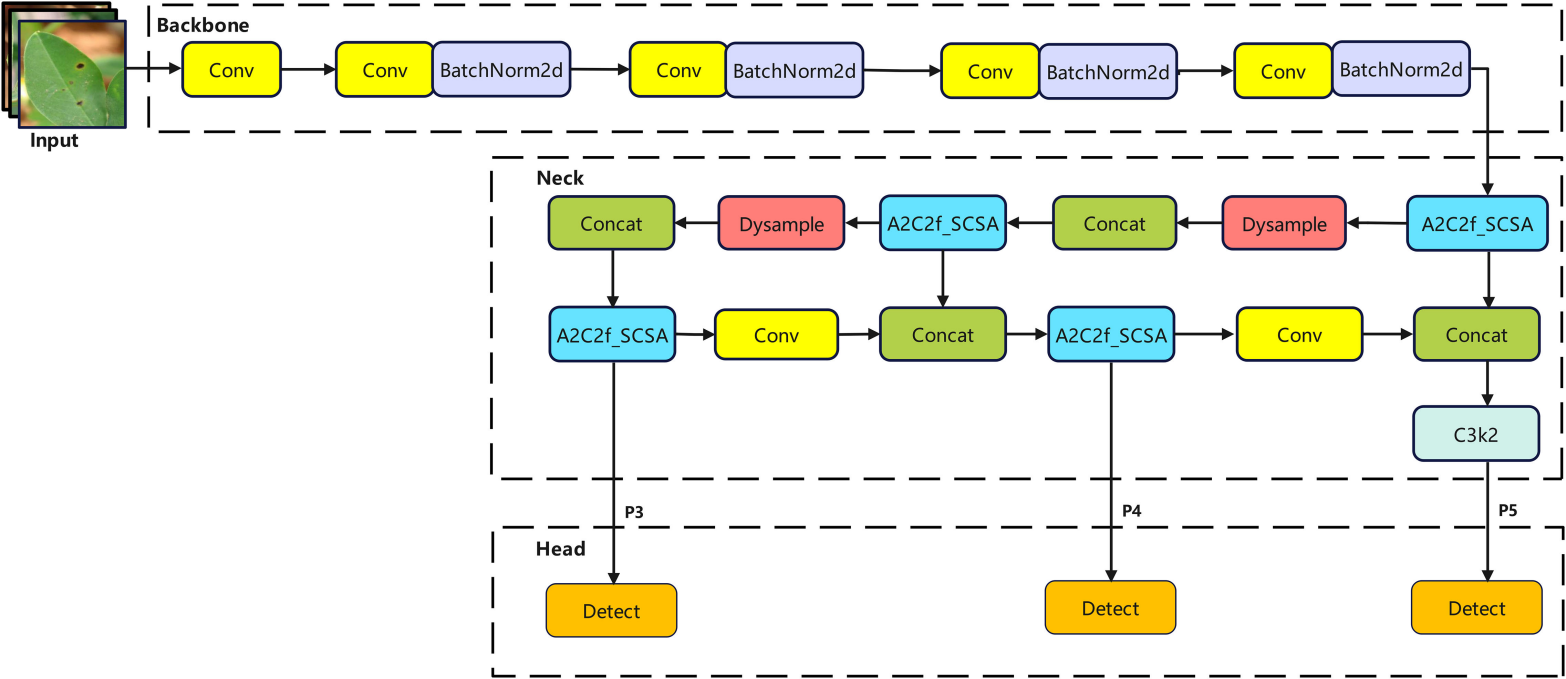
YOLO-SDA structure diagram.

In summary, the improved model (**Figure 10**) enhances feature extraction while optimizing the balance between detection performance and computational efficiency.

#### 2.2.6 Environment and parameter settings

The experimental environment and parameter settings used in this study are detailed in **Table 3**.

**Table 3:** Experimental platform details.

Item	Specification
Operating System	Windows 10 (64-bit)
Processor	12th Gen Intel Core i7-12700F @ 2.10 GHz
Graphics Card	NVIDIA GeForce RTX 3060 Ti (8 GB)
RAM	32 GB
Programming Language	Python 3.11
Deep Learning Framework	PyTorch 2.2.2
CUDA Version	12.1
Batch Size	4
Epochs	150

#### 2.2.7 Evaluation criteria

To evaluate the performance of the improved YOLOv12, Precision (P), Recall (R), mean average precision (mAP), model parameter count (parameters), computational complexity (GFLOPs), and detection frame rate (FPS) were selected as the evaluation metrics for peanut leaf disease detection algorithms.

Precision measures the risk of a model incorrectly predicting negative samples as positive, with the calculation formula shown in Equation 22:

(22) $$P=\frac{\text{TP}}{\text{TP}+\text{FP}}$$

In the formula, TP (True Positive) represents the number of samples correctly predicted as positive by the model; FP (False Positive) represents the number of samples incorrectly predicted as positive by the model (which are actually negative samples).

Recall represents the proportion of correctly detected positive samples by the model relative to the total number of true positive samples, calculated as shown in Equation 23:

(23) $$P=\frac{\text{TP}}{\text{TP}+\text{FN}}$$

In the formula, FN (false negative) represents the number of samples incorrectly predicted as negative by the model (which are actually positive samples).

AP value is the area enclosed by the PR curve and the coordinate axes, used to evaluate model performance. mAP is the average AP across all detection categories, where N represents the number of detection categories. This study detected six peanut leaf diseases, thus N = 6. The formulas for calculating AP and mAP are shown in Equations 24, 25:

(24) $$\text{AP}=\int_{0}^{1}P(R)\;dR$$

(25) $$\text{mAP}=\frac{1}{N}\sum_{i=1}^{N}{\text{AP}}_{i}$$

Additionally, parameters, GFLOPs, and FPS are used as metrics to evaluate the algorithm’s lightweight performance. Parameters refer to the number of parameters, FPS denotes frames per second, and GFLOPs represents “gigaflops,” or the number of floating-point operations per second.

To assess the stability and reproducibility of our experimental results, all experiments were repeated five times with different random seeds. For each evaluation metric (Precision, Recall, and mAP@0.5-0.95), we report the mean and standard deviation across these five runs. The mean $\bar{x}$ and standard deviation (s) are calculated as Equation 26:

(26) $$\bar{x}=\frac{1}{n}\sum_{i=1}^{n}x_{i},\;s=\sqrt{\frac{1}{n-1}{\sum_{i=1}^{n}\left(x_{i}-\bar{x}\right)}^{2}}$$

This approach is widely adopted in machine learning research to quantify the variability of model performance. The standard deviation provides a measure of stability, with smaller values indicating more reliable results.

## 3 Results

### 3.1 Detection results for peanut leaf diseases

**Figure 11:**
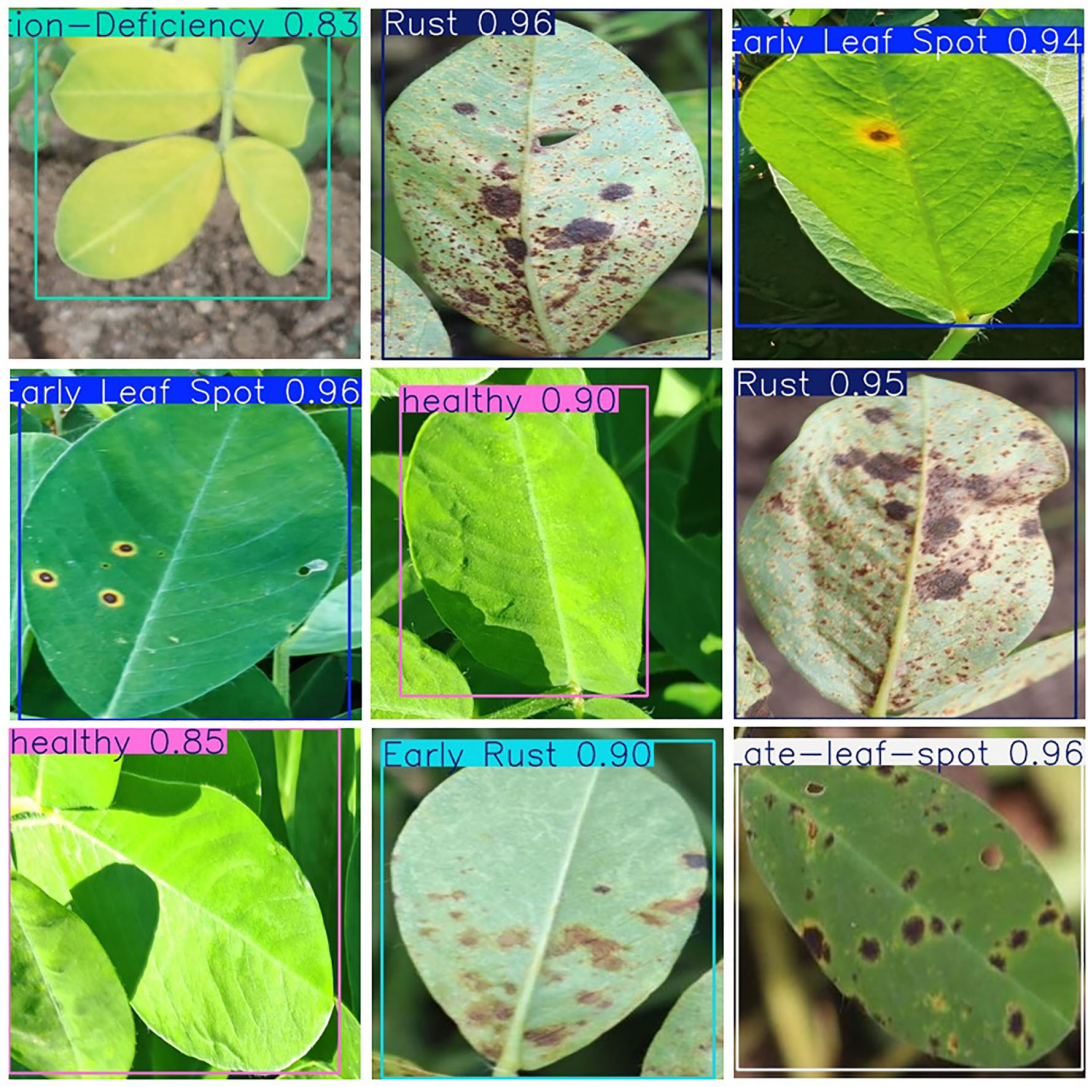
An example image of peanut leaf disease automatically detected by the algorithm.

To evaluate the performance of the improved YOLOv12 model for detecting peanut leaf diseases, we tested it on peanut leaf disease images, classifying and labeling different types of diseases (**Figure 11**).

**Figure 12:**
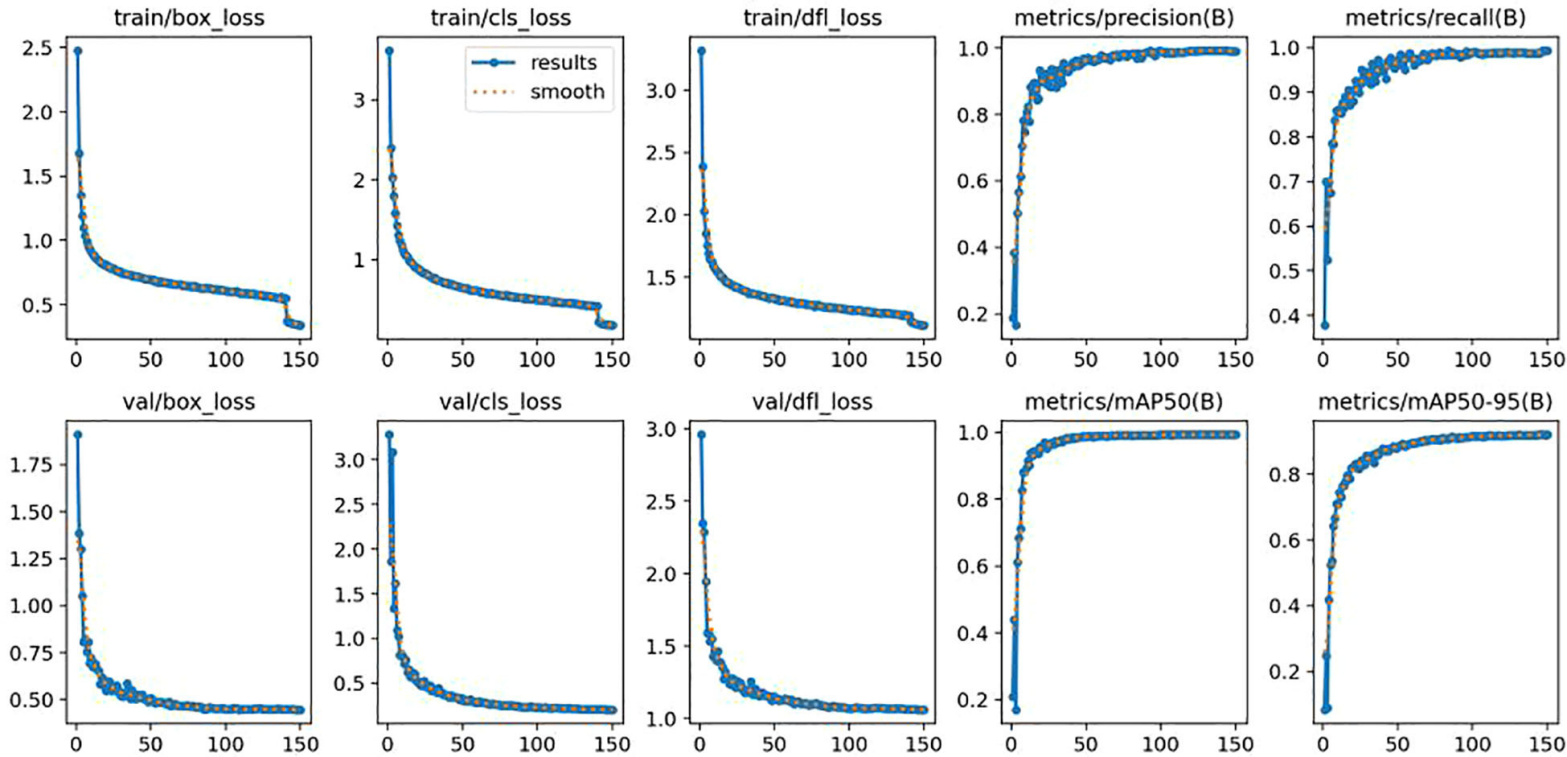
Loss and detection performance curves of improved YOLOv12.

The loss and detection performance curves of the improved YOLOv12 in the training dataset are presented (**Figure 12**). During the first 150 training epochs, loss began to decrease after the 15th epoch, while Precision, Recall, and mAP increased significantly. The loss value then slowly oscillated downward and eventually plateaued. At the 150th training cycle, the model converged. The model achieved remarkable results: at thresholds ranging from 0.5 to 0.95, the detection rate, recall rate, and average accuracy for peanut leaf disease reached 99.3%, 99.1%, and 92.5%, respectively. Concurrently, the improved YOLOv12 model exhibited parameters, model size, and GFLOP values of 1.4MB, 3.1MB, and 4.0G, respectively. The research findings indicate that the improved model demonstrates high accuracy in detecting peanut leaf diseases and is suitable for deployment on resource-constrained devices. This study holds significant importance for advancing the technical methodologies used in peanut leaf disease detection.

### 3.2 Ablation test

**Figure 13:**
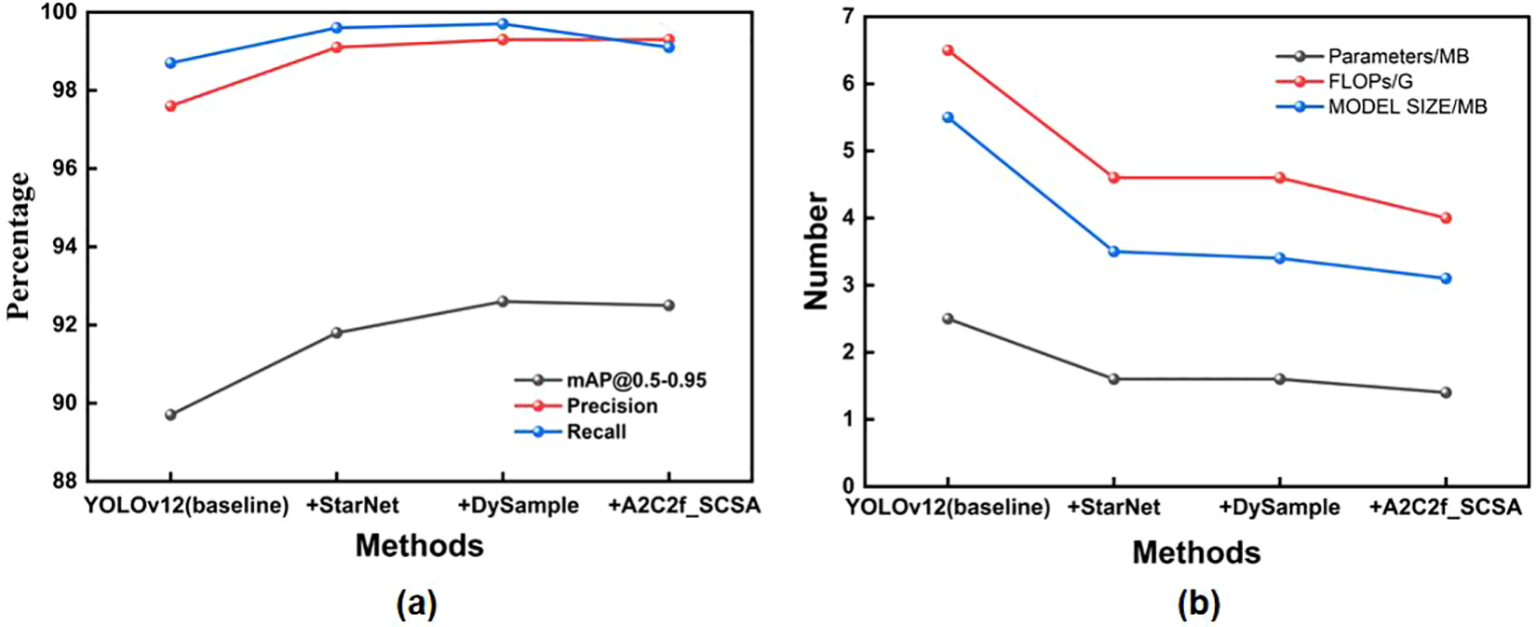
Chart of ablation test performance results. **(A)** mAP@0.5-0.95, Precision and Recall comparison point diagram for ablation test; **(B)** Contrast point chart for Parameters, FLOPs and MODEL SIZE of ablation test.

To validate the impact of each enhancement module on the network model, this study employs YOLOv12 as the baseline and conducts ablation tests on StarNet, DySample, and A2C2f_SCSA. The objective is to achieve lightweight models without sacrificing accuracy. The ablation test results for each model combination are shown in **Table 4**, **Figure 13**. StarNet reduced the parameter count from 2.5MB to 1.6MB and shrunk the model size from 5.5MB to 3.5MB, while increasing the FPS from 357.1 to 416.7, achieving model lightweighting and faster inference speeds. However, its mAP@0.5–0.95 of 91.8% failed to reach peak performance, indicating a need for further accuracy improvements. To compensate for the lack of accuracy, the DySample module was incorporated. This module elevated mAP@ 0.5-0.95 to 92.7% and boosted recall to 99.4%, achieving outstanding detection performance. However, this resulted in a slight increase in parameters and a significant drop in frames per second. To address efficiency concerns, integrating the A2C2f_SCSA module increased FPS to 556.6, reduced FLOPs to 5.5 G, and maintained parameters at 2.5 MB, yet failed to balance lightweight design with high performance; Fusing the three modules—StarNet, DySample, and A2C2f_SCSA—achieved the optimal overall performance. The number of parameters was reduced to 1.4 MB, the model size decreased to 3.1 MB, and FLOPs dropped to 4.0 G, further realizing lightweight optimization. Maintained 92.5% mAP@0.5-0.95 while boosting FPS to 454.5, ensuring efficient inference. Compared to the original YOLOv12, it enhances detection accuracy and inference speed while achieving model lightweighting, making it suitable for small object detection like peanut leaf diseases and complex scenarios.

**Table 4:** Ablation test results.

StarNet	DySample	A2C2f_SCSA	mAP@0.5-0.95/%	Parameters /MB	FLOPs /G	FPS /s−1	Precision/%	Recall/%	Model Size/MB
–	–	–	89.7	2.5	6.5	357.1	97.6	98.7	5.5
✓	–	–	91.8	1.6	4.6	416.7	99.1	99.6	3.5
–	✓	–	92.7	2.6	6.3	256.4	98.7	99.4	5.6
–	–	✓	91.6	2.5	5.5	555.6	98.5	99.0	5.3
✓	✓	–	92.6	1.6	4.6	243.9	99.3	99.7	3.4
✓	–	✓	91.8	1.4	4.0	454.5	99.1	99.5	3.1
**√**	**√**	**√**	**92.5**	**1.4**	**4.0**	**454.5**	**99.3**	**99.1**	**3.1**

*Results in **Table 4** are from a single representative run. For statistical analysis over five runs, please refer to **Table 8**.

The bold values represent the optimal results of each corresponding evaluation metric.

### 3.3 Comparative test

#### 3.3.1 Comparison of different backbone network performance

The backbone networks in YOLOv12 were replaced with StarNet, PoolFormer (Yu et al., 2022; Yu et al., 2024), and FasterNet (Chen et al., 2023), respectively. StarNet focuses on synergistic optimization of lightweight design and performance, PoolFormer represents a classic pooling-based lightweight architecture, while FasterNet centers its design around high inference efficiency. The performance metrics of StarNet, PoolFormer, and FasterNet are compared with those of the original YOLOv12 backbone network. The comparison results are shown in **Table 5**. In terms of lightweight performance, StarNet achieves the smallest parameter count and model size, reducing these metrics by 36% and 36.4% compared to YOLOv12, by 87.4% and 86.4% compared to PoolFormer, and by 52.9% and 50.7% compared to FasterNet. In computational efficiency, it achieves the lowest FLOPs, reducing by 29.2% compared to YOLOv12, 86% compared to PoolFormer, and 46.5% compared to FasterNet. Its FPS outperforms YOLOv12 and PoolFormer by 16.7% and 217% respectively, matching FasterNet’s performance. In detection accuracy, it achieved the highest Precision and Recall at 99.1% and 99.6%, respectively, mAP@0.5-0.95 outperformed YOLOv12 and PoolFormer by 2.1 and 4.5 percentage points, while only falling 0.1 percentage points short of FasterNet. These results substantiate our initial motivation for choosing StarNet: it enhances local feature extraction while maintaining a lightweight profile, achieving an optimal balance between lightweight architecture, efficient inference, and high accuracy.

**Table 5:** Performance comparison of different backbone networks.

Backbone	Parameters/MB	FLOPs/G	MODEL SIZE/MB	FPS /s−1	Precision/%	Recall/%	mAP@0.5-0.95/%
YOLOv12	2.5	6.5	5.5	357.1	97.6	98.7	89.7
**StarNet**	**1.6**	**4.6**	**3.5**	**416.7**	**99.1**	**99.6**	91.8
PoolFormer	12.7	32.8	25.7	131.6	96.9	97.8	87.3
FasterNet	3.4	8.6	7.1	416.7	98.6	99.4	91.9

The bold values represent the optimal results of each corresponding evaluation metric.

#### 3.3.2 Performance comparison of optimized neck network performance

Selecting an appropriate neck network can effectively address the challenges of detecting small or damaged targets. To validate the effectiveness of DySample in peanut leaf disease detection, this paper compares YOLOv12 with DySample, Semantics and Detail Infusion (SDI) (Zhou and Zhou, 2024), and Rectangular Calibration Method (RCM) (Ni et al., 2025). The comparison results are shown in **Table 6**. Results indicate that DySample achieves a mAP@0.5–0.95 of 92.7%, representing a 3-percentage-point improvement over YOLOv12 and outperforming both SDI and RCM. It also demonstrates the highest Precision and Recall, surpassing YOLOv12 by 1.1 and 0.7 percentage points, respectively. Meanwhile, its parameters and model size increase only slightly, with FLOPs slightly lower than YOLOv12, ensuring manageable resource consumption. Although the FPS decreases marginally, its detection performance demonstrates a significant advantage. This approach provides an effective pathway for enhancing the overall performance of object detection tasks, demonstrating significant advantages, particularly in complex scenarios. These findings confirm that DySample’s dynamic upsampling is superior to other fusion methods for reconstructing fine-grained lesion details, thereby justifying its selection for the Neck network.

**Table 6:** Influence of different enhancement feature fusion on detection performance.

Neck Function	Parameters/MB	FLOPs/G	Model Size /MB	FPS /s−1	Precision/%	Recall /%	mAP@0.5-0.95/%
YOLOv12	2.5	6.5	5.5	357.1	97.6	98.7	89.7
**DySample**	**2.6**	**6.3**	**5.6**	**256.4**	**98.7**	**99.4**	**92.7**
SDI	2.6	6.8	5.7	204.1	98.1	99.0	92.2
RCM	3.0	7.0	6.5	344.8	97.9	99.4	92.4

The bold values represent the optimal results of each corresponding evaluation metric.

#### 3.3.3 Performance comparison of different attention mechanisms

To evaluate multiple attention mechanisms, YOLOv12 was adopted as the baseline model. By introducing different attention mechanisms into the model, their effectiveness was demonstrated through comparisons across multiple evaluation metrics. The comparative experimental results are shown in **Table 7**.In the peanut leaf disease detection task, after evaluating YOLOv12, A2C2f_SCSA, MCAttn (Dai et al., 2024; Xu et al., 2025b), SimAM, CA (Hou et al., 2021; Zeng et al., 2025), and GAM (Li et al., 2024), A2C2f_SCSA emerged as the relatively optimal choice.A2C2f_SCSA achieved mAP@0.5–0.95 of 91.6%, Precision of 98.5%, and Recall of 99.0%. All three core accuracy metrics outperform YOLOv12 and significantly surpass MCAttn and SimAM. This model accurately identifies peanut leaf disease features, effectively reducing misclassifications and missed detections, thereby meeting the core accuracy requirements for peanut leaf disease detection in agricultural scenarios. Although A2C2f_SCSA’s parameters, FLOPs, and model size are not the lowest among all mechanisms, its resource consumption is comparable to YOLOv12. This ensures that training and deployment on mobile devices in field settings do not incur excessive computational burdens or storage pressures. In contrast, while MCAttn and SimAM feature fewer parameters and lower computational complexity, their detection accuracy falls far short of meeting the precise identification requirements for peanut leaf diseases. Although CA achieves a slightly higher mAP@0.5-0.95, it suffers from increased parameter counts and reduced inference speed. GAM’s mAP@0.5-0.95 shows only a marginal improvement over YOLOv12, offering no practical advantage. The results clearly demonstrate that the synergistic spatial-channel attention of SCSA is more effective at suppressing background noise and enhancing lesion features than other mechanisms, validating its integration into the A2C2f module.

**Table 7:** Effects of various attention mechanisms on detection performance.

Attention Mechanisms	Parameters/MB	FLOPs/G	Model Size /MB	FPS /s−1	Precision /%	Recall/%	mAP@0.5-0.95/%
YOLOv12	2.5	6.5	5.5	357.1	97.6	98.7	89.7
**A2C2f_SCSA**	2.5	5.5	5.3	**555.6**	**98.5**	**99.0**	**91.6**
MCAttn	1.8	5.0	3.9	555.6	88.4	97.8	70.2
SimAM	1.8	5.0	3.9	526.3	93.6	97.4	75.9
CA	2.6	6.3	5.6	384.6	98.5	98.8	92.4
GAM	2.4	5.7	5.1	476.2	97.2	98.8	89.8

The bold values represent the optimal results of each corresponding evaluation metric.

#### 3.3.4 Comparison of different algorithms

**Figure 14:**
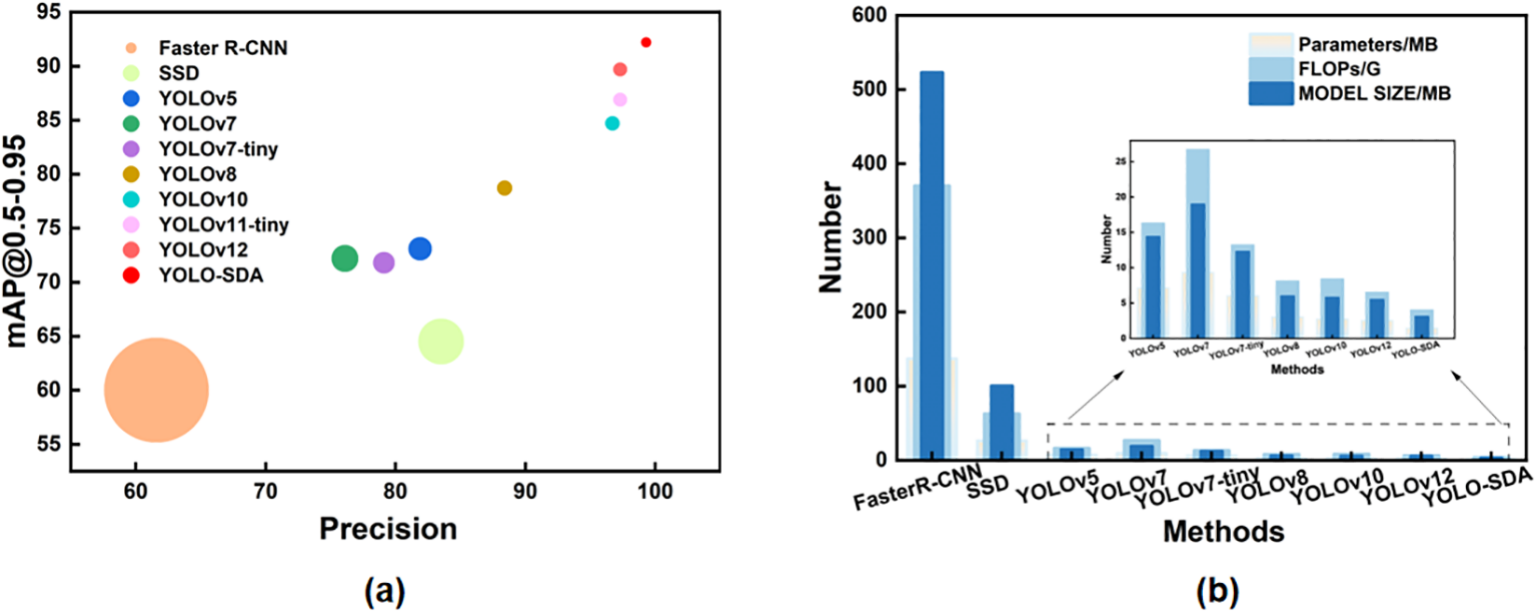
Model analysis and comparison results. (A) is the comparison between mAP@0.5-0.95 and Precision, and the size of the circle indicates the number of parameters; (C) is the nested histogram of Parameters, FLOPs and Model Size.

To further demonstrate the superiority of the improved YOLOv12 in detecting leaf diseases on peanuts, we compared the Precision, Recall, mAP@0.5-0.95, Parameters, Model Size, and GFLOPs of various trained models. Algorithms were compared using commonly used series models, including SSD, Faster R-CNN, YOLOv5, YOLOv7, YOLOv7-tiny, YOLOv8, YOLOv10, YOLOv11-tiny, and YOLOv12. As shown in **Table 8**, **Figure 14**, the proposed YOLO-SDA model achieves significant lightweight improvements while maintaining high detection accuracy. Compared to YOLOv12, the smallest model in terms of size, Parameters decreased from 2.5MB to 1.4MB, a reduction of 44%; GFLOPs dropped from 6.5G to 4.0G, a decrease of 38.5%; Model Size shrank from 5.5MB to 3.1MB, a reduction of 43.6%. In terms of detection performance, YOLO-SDA achieves a mean Precision of 99.3% ± 0.1%, Recall of 99.0% ± 0.1%, and mAP@0.5-0.95 of 92.2% ± 0.2%. This represents improvements of 2.0%, 0.7%, and 2.5% over YOLOv12 (97.3% ± 0.5%, 98.3% ± 0.5%, and 89.7% ± 0.4%), respectively.

**Table 8:** Experimental results of model analysis and comparison.

Methods	Parameters /MB	FLOPs /G	Model Size /MB	Precision/%	Recall/%	mAP@0.5-0.95/%
Faster R-CNN	137.01	370.21	523.0	61.6 ± 0.5	72.0 ± 0.4	60.0 ± 0.6
SSD	26.29	62.7	100.3	83.5 ± 0.3	62.0 ± 0.5	64.5 ± 0.4
YOLOv5	7.1	16.3	14.4	81.9 ± 0.2	75.5 ± 0.1	73.1 ± 0.1
YOLOv7	9.3	26.7	19.0	76.1 ± 0.2	76.8 ± 0.3	72.2 ± 0.1
YOLOv7-tiny	6.0	13.2	12.3	79.1 ± 0.3	77.7 ± 0.2	71.8 ± 0.1
YOLOv8	3.0	8.1	6.0	88.4 ± 0.2	92.0 ± 0.5	78.7 ± 0.2
YOLOv10	2.7	8.4	5.8	96.7 ± 0.2	96.9 ± 0.3	84.7 ± 0.2
YOLOv11-tiny	2.5	6.4	5.4	97.3 ± 0.3	98.4 ± 0.3	86.9 ± 0.7
YOLOv12	2.5	6.5	5.5	97.3 ± 0.5	98.3 ± 0.5	89.7 ± 0.4
**YOLO-SDA**	**1.4**	**4.0**	**3.1**	**99.3 ± 0.1**	**99.0 ± 0.1**	**92.2 ± 0.2**

*Results are reported as mean ± standard deviation over five independent runs. The best results for each metric are highlighted in bold.

The bold values represent the optimal results of each corresponding evaluation metric.

Compared to other algorithms, the parameters, FLOPs, and model size of YOLO-SDA are significantly lower than all other versions. Notably, the small standard deviations across all models (≤0.5% for Precision and Recall, ≤0.7% for mAP) confirm the stability and reproducibility of our experimental results. Among all compared models, YOLO-SDA consistently achieves the highest mean values across all evaluation metrics: Precision (99.3% ± 0.1%), Recall (99.0% ± 0.1%), and mAP@0.5-0.95 (92.2% ± 0.2%), further demonstrating its superior performance.

#### 3.3.5 Disease identification system image interface

Field research findings indicate that peanut farmers in Xiachangmao Village, Huashan Town, Xingcheng City, Liaoning Province, China, exhibit inaccuracies in identifying peanut leaf diseases, lack clarity in disease classification, fail to implement timely control measures, and employ unscientific prevention methods. They predominantly rely on subjective experience to diagnose diseases and conduct chemical control, resulting in untimely application of pesticides, inaccurate selection of chemical types, and unscientific dosage management.

**Figure 15:**
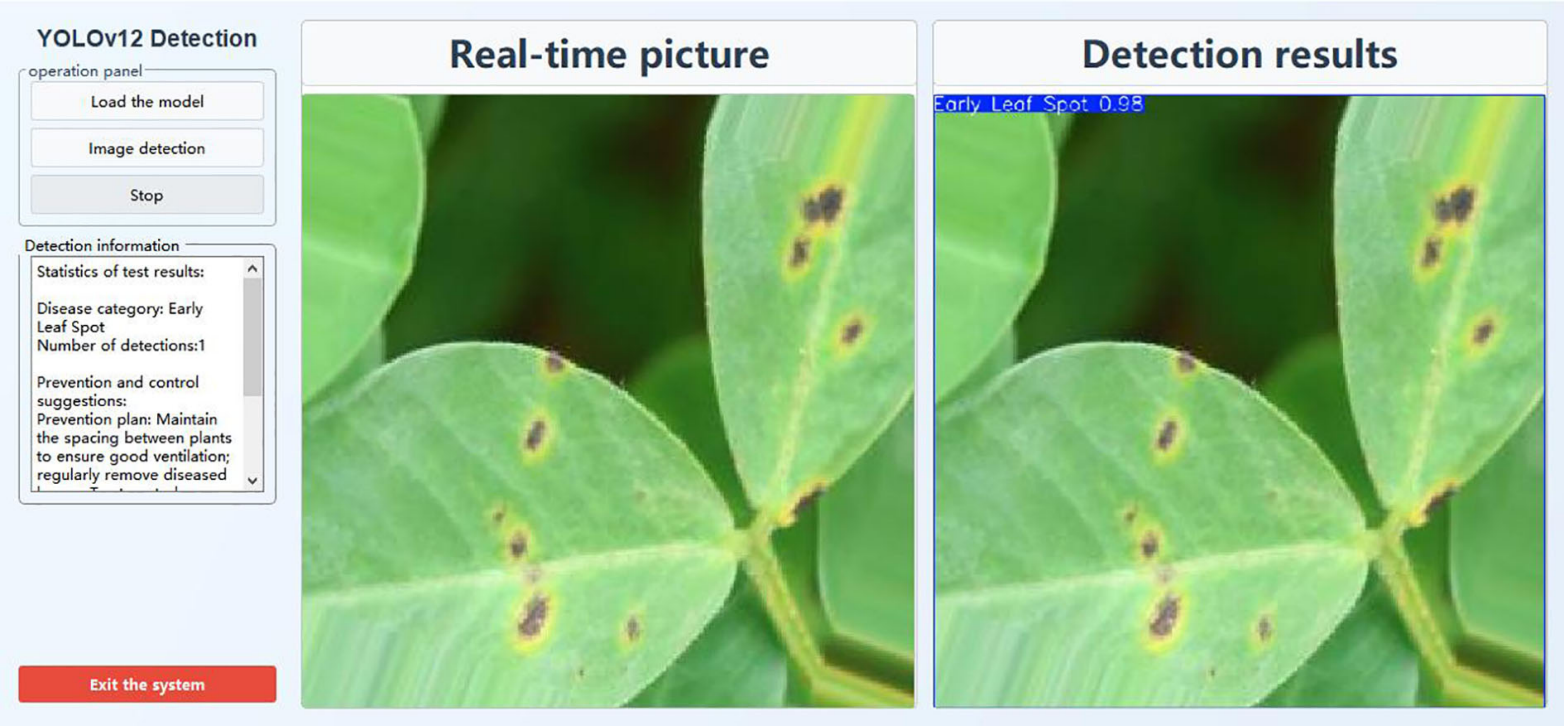
Image interface of disease identification system.

To facilitate the implementation of the improved crop disease identification model in agricultural production settings, this study developed a lightweight disease identification system interface based on the Python PyQt5 framework. Simple and easy to operate, peanut farmers can use this identification system to promptly and accurately identify peanut disease outbreaks and provide targeted control measures (**Figure 15**). Click on an image on the left side of the interactive interface to initiate image recognition. The lower left corner displays the identified disease type along with corresponding control recommendations, while the right side shows real-time footage and detection results.

In the current prototype, these recommendations are formulated based on general agronomic knowledge and expert experience in peanut disease management. They are provided as static reference information to illustrate the system’s potential, rather than dynamically generated, context-aware advice.

We acknowledge that for practical deployment, recommendations must be tailored to specific disease types, severity levels, and field conditions (e.g., weather, growth stage). This limitation will be addressed in future work by: (1) developing a dynamic knowledge base that links detection results with up-to-date, region-specific agronomic guidelines; (2) integrating environmental data (e.g., weather forecasts, soil moisture) to generate more precise, context-aware recommendations; and (3) collaborating with agricultural extension experts to validate and refine the recommendation logic. Additionally, future iterations of the system will incorporate disease severity information once severity grading capabilities are developed, enabling more precise and actionable control strategies.

Testing demonstrates that this system reliably achieves its predefined functional objectives of accurate disease identification and serves as a foundation for future decision-support tools. It not only accurately identifies types of peanut leaf diseases but also provides scientifically grounded prevention references, offering preliminary decision support for peanut field disease management.

### 3.4 Heatmap visualization analysis

**Figure 16:**
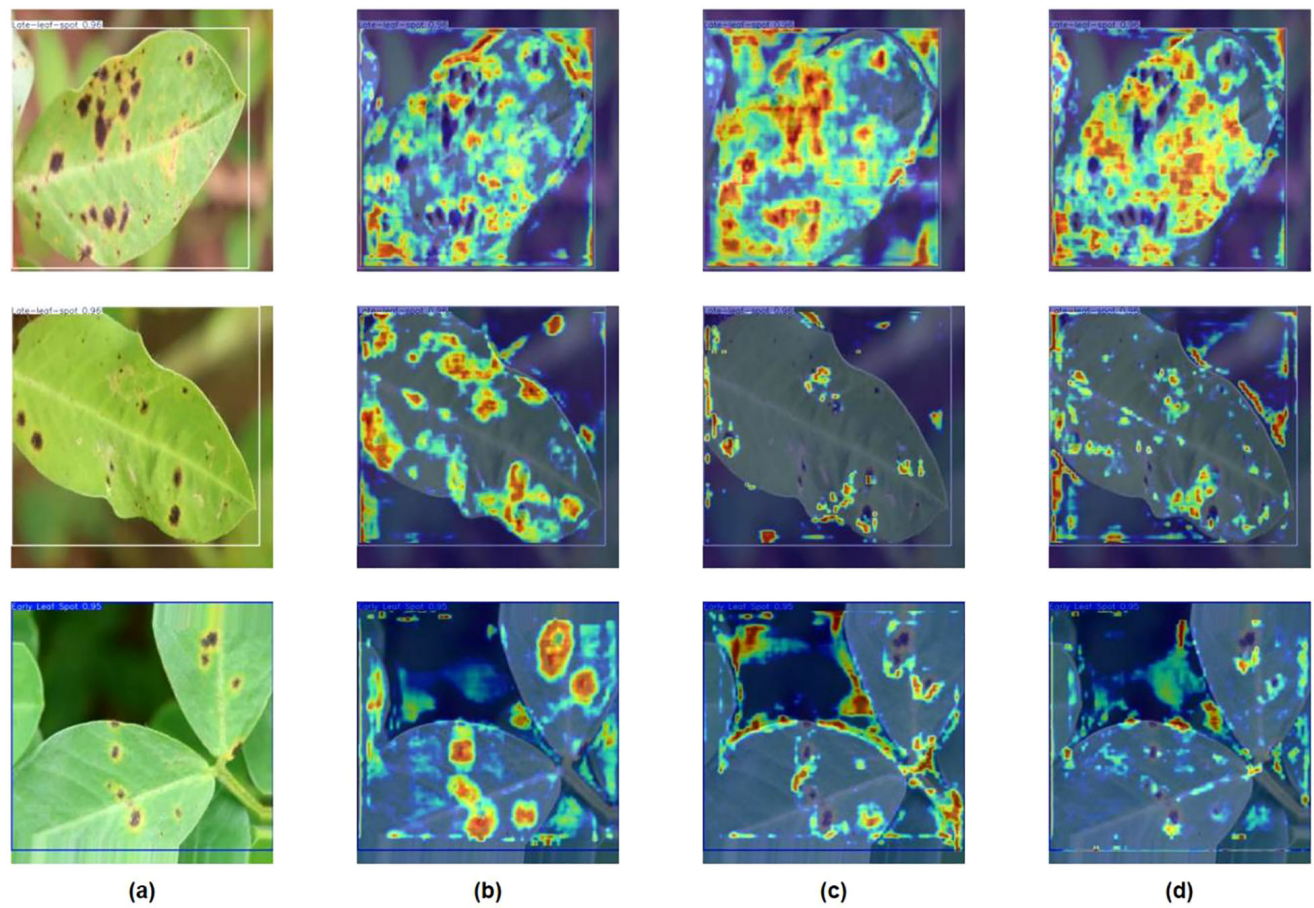
Uses various methods to visually analyze the improved YOLOv12 network. The color intensity in the thermogram corresponds to the importance of predicting the characteristics of peanut leaf diseases. The visual description in the figure is as follows. **(A)** original image, **(B)** GradCAM, **(C)** RandomCAM and **(D)** XGradCAM.

To provide a clearer and more intuitive analysis of the improvement effects of the YOLO-SDA model, visualization analysis was conducted using GradCAM (Chen et al., 2024; Selvaraju et al., 2020), RandomCAM (Yuan et al., 2025), and XGradCAM (Fu et al., 2020) heatmaps. The intensity of colors in the generated heatmaps indicates the locations where the model focuses its attention, highlighting specific areas on the leaf surface affected by disease (**Figure 16**). The first column (A) shows the original image, while the second to fourth columns (B, C, and D) display the heatmaps generated by GradCAM, RandomCAM, and XGradCAM, respectively. It is evident that the heatmaps generated by YOLO-SDA focus more intently on diseased areas. This concentration enhances the capture of multi-scale features within images, thereby improving detection accuracy—particularly when handling small objects—and reducing the likelihood of missed detections.

### 3.5 Mobile detection device and visual analysis

**Figure 17:**
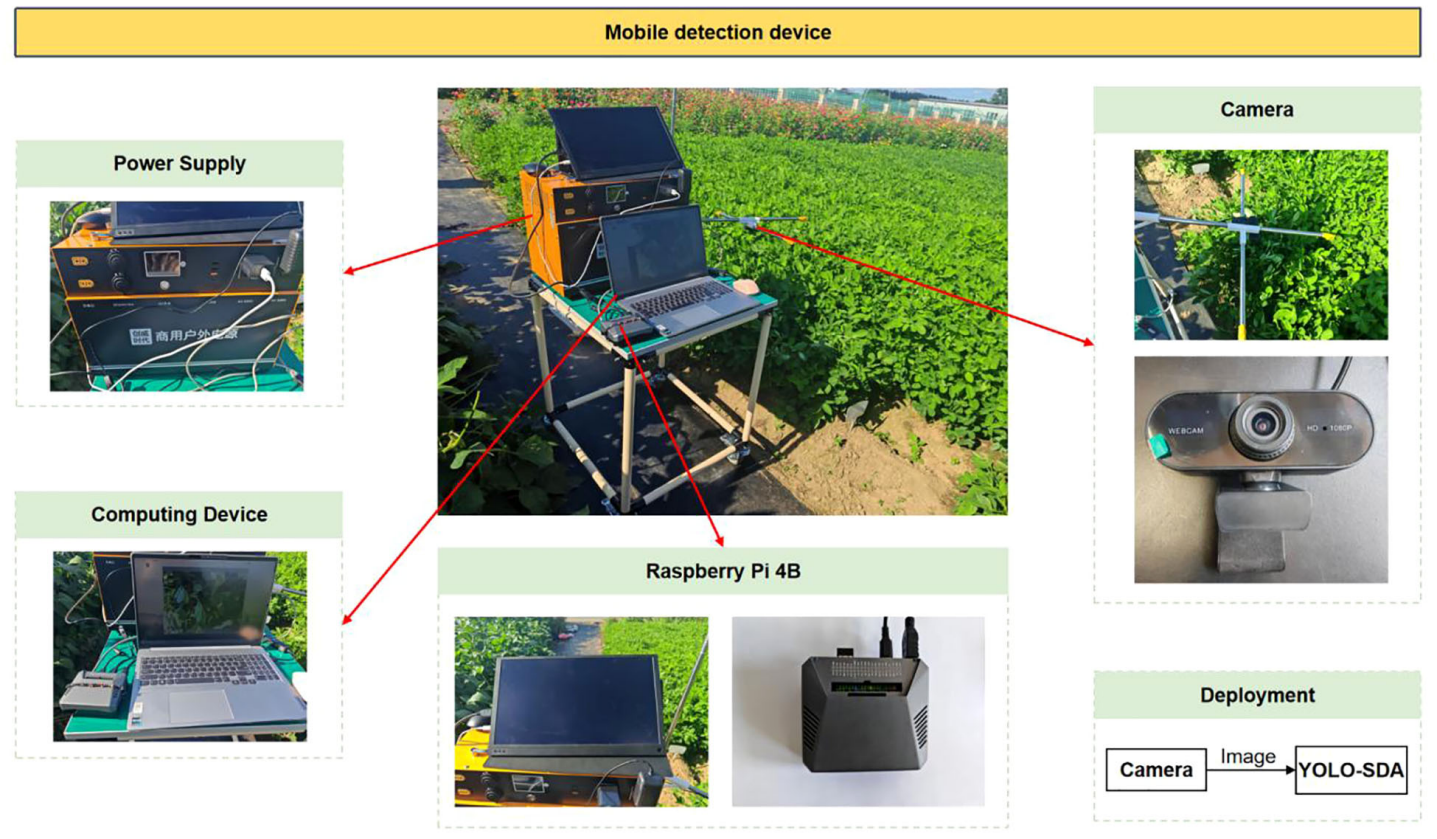
Mobile detection device.

**Figure 18:**
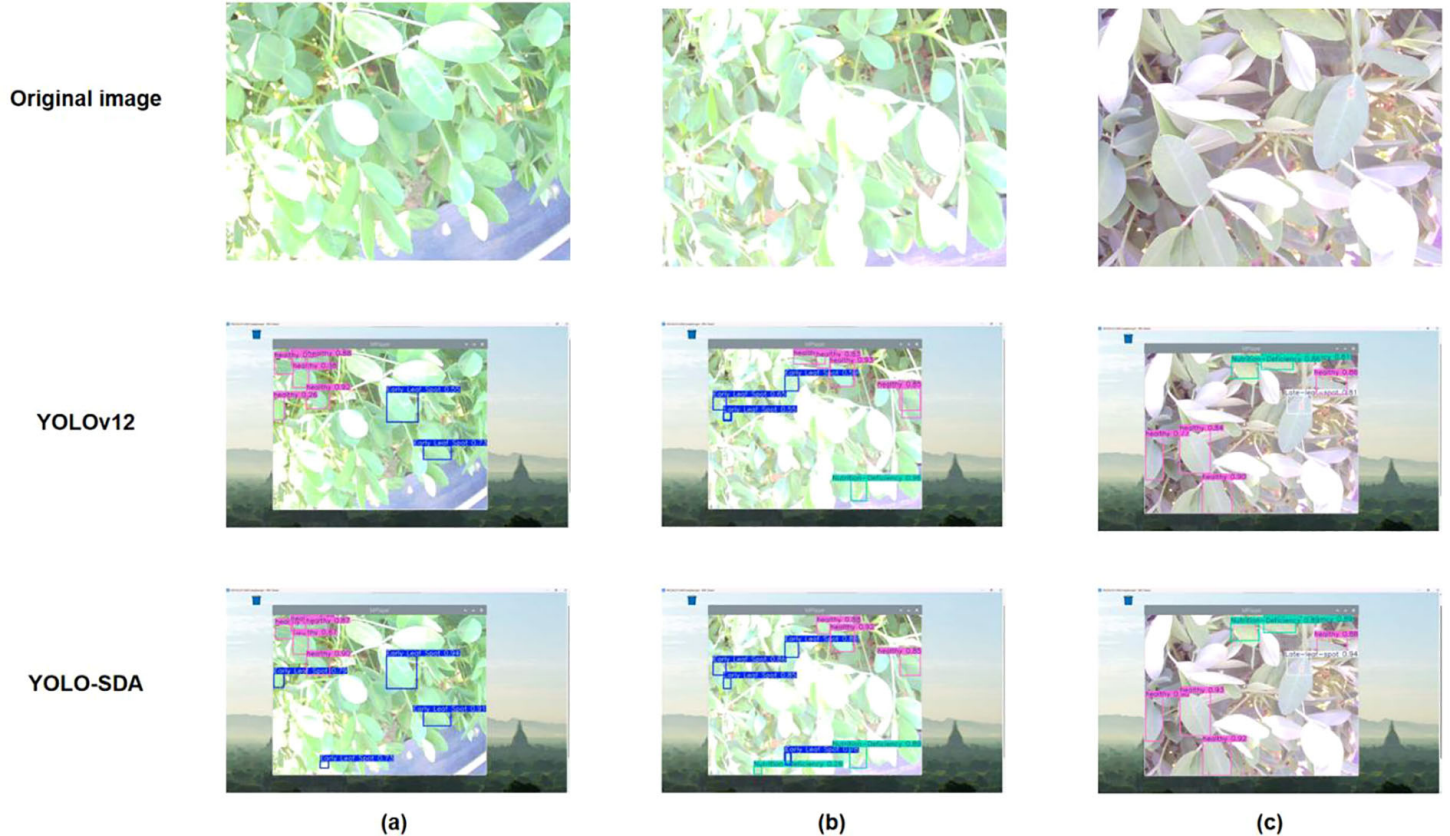
Analysis of prediction results of field experiments.

The improved YOLO-SDA algorithm was deployed on a Raspberry Pi 4B and tested using image data captured by a camera. The Raspberry Pi 4B features a 1.5GHz 64-bit quad-core ARM Cortex-A72 CPU, a 64GB microSDXC card, dual-band 2.4GHz and 5.0GHz Wi-Fi, and Bluetooth 5.0 technology. To enable dynamic monitoring of peanut leaf diseases, a mobile detection device was deployed in the field for practical testing. This device utilizes a Raspberry Pi 4B as its embedded hardware platform and incorporates a vehicle-mounted frame, camera, portable power supply, Raspberry Pi, and computer. The camera mount is positioned 80 centimeters above the blade height. The device was deployed in the field for practical testing (**Figure 17**), and detection results are shown in **Figure 18**.

The first row shows the original image, the second row displays the detection results from YOLOv12, and the third row presents the detection results from the improved YOLO-SDA. Compared to YOLOv12, YOLO-SDA enhances detection accuracy and reduces false negative rates in scenarios such as lesion occlusion, multi-object detection, and dense foliage. The inference time per image is only 62.5 milliseconds.

## 4 Discussion

### 4.1 Advantages and limitations

If peanut leaf diseases are not detected promptly, they can cause leaf drop and reduce photosynthesis, thereby affecting yield. Traditional methods for monitoring peanut leaf diseases typically rely on manual visual inspection and field surveys. With the advancement of computer vision through machine learning and deep learning research, a combined approach of hyperspectral imaging and computer vision has been adopted for leaf disease detection. However, as the distance to the target increases, issues such as blurring and difficulty in identifying small targets arise. To address these challenges, this study proposes an optimized lightweight YOLO-SDA detection algorithm.

This model integrates improvements from Starnet, DySample, and A2C2f_SCSA. These enhancements enable lightweight implementation without compromising detection accuracy, while simultaneously increasing detection speed and reducing the number of parameters and model size. Through comparative and ablation experiments and analysis, the proposed lightweight YOLO-SDA network model demonstrates high detection accuracy and low computational complexity for peanut leaf disease detection. This makes it suitable for achieving a balance between compactness and accuracy in intelligent peanut leaf disease detection devices.

Although YOLO-SDA demonstrates high accuracy and lightweight real-time deployment potential on the Raspberry Pi 4B, its generalization capability and practical application value remain under-validated, primarily constrained by two factors: cross-dataset generalization experiments have not been conducted due to the lack of a uniformly annotated public peanut leaf disease dataset; furthermore, while field trials show promising prospects, their scale is limited (single location, restricted timeframe). Future work will address these limitations through the following approaches: (1) Collecting multi-regional data across different seasons and diverse peanut-growing areas to enhance dataset diversity and model generalization; (2) Conducting long-term field trials under broader environmental conditions (e.g., varying light, weather, and growth stages); (3) Integrating RGB imaging with hyperspectral data, as the latter captures unique spectral signatures of different diseases, enabling differentiation even among visually similar colors; (4) Developing multi-angle imaging systems to capture leaf images from multiple perspectives, enabling the model to learn more robust three-dimensional structural features of lesions and thereby enhance recognition capabilities.

Furthermore, we acknowledge that the current study focuses only on disease type classification and does not yet address disease severity grading—a crucial factor for practical agricultural applications. Accurate severity assessment enables farmers to determine appropriate treatment urgency and pesticide dosage, which is essential for precision agriculture. Future work will extend YOLO-SDA to incorporate disease severity quantification by integrating a semantic segmentation branch into the network to precisely segment lesion areas and compute lesion-to-leaf pixel ratios.

In addition to these dataset- and imaging-focused improvements, several broader challenges warrant investigation. From a technical perspective, improving model interpretability (e.g., via enhanced visualization techniques) would build trust with end-users, and exploring few-shot learning could enable rapid adaptation to newly emerging diseases with limited samples. From an application perspective, integrating the detection model with precision agriculture actuators, such as drones for targeted pesticide spraying, would create a closed-loop disease management system. Finally, developing a more user-friendly mobile application with offline capabilities is crucial for ensuring the technology is accessible and practical for farmers in the field.

These combined efforts will further validate the model’s robustness and advance its transition from a research prototype to a practical precision agriculture tool.

## 5 Conclusions

To address the challenge of accurately detecting similar leaf diseases and incomplete targets in complex environments, this study collected images through both field sampling and automated network-based methods. Following image annotation, a target detection dataset was established, encompassing six common leaf diseases affecting peanuts. Building upon this foundation, we improved the YOLOv12 model and proposed a novel disease detection algorithm, YOLO-SDA, achieving accurate detection of six types of peanut leaf diseases.

This algorithm achieves improved detection accuracy without compromising performance by integrating Starnet, DySample, and A2C2f_SCSA into a lightweight framework. Replacing Backbone with the lightweight StarNet effectively reduced the number of parameters; substituting the original upsampling module with the lightweight and efficient DySample dynamic module significantly enhanced the ability to capture and recognize small target lesions with diverse features; and optimizing feature weight allocation using A2C2f_SCSA improved the model’s detection capability for peanut leaf diseases across different scenarios.

Experimental results show that YOLO-SDA achieves detection accuracy and recall rates of 99.3% and 99.0%, respectively, with mAP@0.5-0.95 at 92.2%. Compared to the original YOLOv12, this represents improvements of 2.0 and 2.5 percentage points in precision and mAP@0.5-0.95, respectively. Additionally, the number of parameters, model size, and GFLOPs were reduced by 44%, 38.5%, and 43.6%, respectively. The findings of this study provide an important reference for the development of intelligent peanut leaf detection equipment.

## Data Availability

The original contributions presented in the study are included in the article/supplementary material. Further inquiries can be directed to the corresponding authors.
